# Health Effects of Various Edible Vegetable Oil: An Umbrella Review

**DOI:** 10.1016/j.advnut.2024.100276

**Published:** 2024-07-23

**Authors:** Phooi Tee Voon, Choon Ming Ng, Yen Teng Ng, Yen Jun Wong, Sia Yen Yap, Siew Lian Leong, Xiou Shuang Yong, Shaun Wen Huey Lee

**Affiliations:** 1 Product Development and Advisory Services Division, Nutrition Unit, Malaysian Palm Oil Board, Selangor, Malaysia; 2 School of Pharmacy, Monash University Malaysia, Subang, Selangor, Malaysia; 3 International Medical University, Kuala Lumpur, Malaysia

**Keywords:** tropical oil, nontropical oil, cardiovascular, lipid, umbrella review

## Abstract

Vegetable oils, derived from diverse sources such as seeds, nuts, and some fruits, play a significant role in dietary health. However, the current evidence on the health effects of different types of vegetable oil consumption remains controversial. This umbrella review aims to synthesize evidence from systematic reviews and meta-analyses to assess the health outcomes associated with various vegetable oils. A comprehensive literature search was performed up to 31 July, 2023 on 12 databases for studies examining the association of different vegetable oils with health outcomes in adults. Data was extracted independently by 2 authors, with evidence strength assessed using the grading of recommendations, assessment, development, and evaluation criteria. A total of 48 studies, including 206 meta-analyses, were included. Moderate to very low certainty evidence showed that monounsaturated and polyunsaturated fatty acids such as canola oil, virgin olive oil, and rice bran oil are beneficial in reducing serum total cholesterol and low-density lipoprotein (LDL) concentrations. Conversely, low to very low certainty evidence suggests that oils high in saturated fats, such as coconut oil and palm oil, increase total cholesterol and LDL concentrations but also raise high-density lipoprotein concentrations. Very low certainty evidence showed the consumption of olive oil, sesame oil, and coconut oil could improve blood sugar control. Low certainty evidence showed olive oil consumption reduced risk of breast, digestive, and other cancers. Moderate to very low certainty evidence suggested that canola oil and sesame oil consumption reduced body weight. The consumption of vegetable oil appears to offer different health benefits, with summary estimates indicating beneficial effects on reducing lipid concentrations, especially with monounsaturated and polyunsaturated rich oils when consumed in recommended amounts. Future research should focus on long-term studies and comprehensive dietary assessments to better understand the health impacts of vegetable oils, providing a basis for informed dietary recommendations.

This study was registered at PROSPERO as CRD42021239210.

**Statement of Significance**

We present a comprehensive and up-to-date overview of the effects of various vegetable oil consumption and highlight the distinct benefits and harms of MUFA, PUFA, and SFAs-rich oils. This work underscores the necessity of differentiating between types of vegetable oils, such as virgin, compared with refined, due to their distinct health effects, an aspect not thoroughly addressed in prior studies.

## Introduction

Vegetable oils are a heterogenous group of oils that are extracted from plant seeds (e.g., flaxseed oil, canola oil), nuts (e.g., peanut oil), flesh of fruits (e.g., palm oil, olive oil) as well as bran (e.g., rice bran oil) [1]. In the past decades, studies on dietary fats and oils have gained interest. The diet-heart hypothesis suggests that the deposition of cholesterol in the arterial wall can be reduced by the modification of dietary fats, wherein saturated fats are replaced with vegetable oils rich in unsaturated fats [2,3]. This subsequently slows the progression of atherosclerosis and coronary artery disease, and the survival rate can be improved. Dietary guidelines recommend the substitution of saturated fats with unsaturated fats, including vegetable oils, to improve health outcomes. This is consistent with the beneficial effects observed in cardiometabolic health and longevity found in the prospective cohort studies using canola oil and olive oil [4,5]. Nevertheless, there has been growing controversy on the consumption of vegetable oils on health outcomes, including the incidence of cardiovascular diseases (CVDs), all-cause deaths, metabolic syndrome, and nonfatal cardiovascular events. Some randomized controlled trials (RCTs) revealed that the benefits of vegetable oils in lowering serum cholesterol concentrations did not translate into better clinical outcomes in terms of disease risks and survival [2,3].

Studies have shown that the benefits or harms of vegetable oils are highly dependent on the fatty acid profile in terms of the type and fraction of fatty acids present [6]. For instance, oils predominantly composed of monounsaturated oleic acids, such as olive oil, canola oil, peanut oil, and rice bran oil, have been linked with anti-inflammation and lipid-lowering properties [[7], [8], [9]]. In addition, research has documented the role of oils containing PUFAs such as the n–3 α-linolenic acid (ALA) and *n–6* linoleic acid (LA) including flaxseed oil (∼50% ALA), traditional sunflower oil (∼60–70% LA), soybean oil (∼51% LA), sesame oil (∼43% LA) and peanut oil (∼30% LA) [1]. Both ALA and LA are important as they provide essential fatty acids that cannot be synthesized by the human body, along with reported cardioprotective effects [10]. On the contrary, palm oil contains a balanced ratio of SFA and unsaturated fatty acids (∼50% palmitic and stearic acid, along with ∼40% oleic acid) [11]. The palmitic acid content contributes to the heat stability of palm oil, yet the high degree of saturation has been associated with increased LDL cholesterol [12]. Similarly, coconut oil mainly comprises SFAs, particularly the predominant medium-length saturates, lauric acid. Although the use of coconut oil has garnered interest for its purported benefits related to lauric acid, these remained inconclusive given that lauric acid may not biologically act as medium-chain triglycerides, along with the presence of other long-chain SFAs in coconut oil [13,14].

Considering the association between nutritional habits and overall health, there is now a suggestion to shift from focusing on the effects of individual fatty acids to edible cooking oils as a whole concerning health outcomes [6,7]. Several reviews have studied the effects of various edible oils on health. For instance, a significant reduction in the risk of CVD events and weight gain was reported on olive oil through observational studies and RCTs [8]. Another systematic review found some improvement in fasting blood glucose and insulin sensitivity with the consumption of flaxseed oil.

To date, there is no comprehensive overview of studies comparing the various health effects of edible vegetable oils, suggesting that this aspect needs to be revisited. We aimed to summarize the existing evidence comparing the health effects of vegetable oils on health outcomes, including blood pressure, blood lipid concentrations, and glucose concentrations, to serve as a reference for future researchers in this area.

## Methods

The study was registered in PROSPERO (CRD42021239210). The systematic literature search adhered to the guidelines outlined in the PRISMA.

### Literature search and selection criteria

A systematic literature search was performed up to 31 July, 2023 using the following databases: PubMed, Embase, PsycINFO, Database of Abstracts of Review of Effectiveness, Health Technology Assessment databases and reports, National Health Service (NHS) Economic Evaluation Database, HealthSTAR, BIOSIS, Science Citation Index, Cochrane Central Register of controlled trials, CINAHL Plus and Allied and Complementary Medicine Database. The search focused on systematic reviews and meta-analyses investigating the health effects of vegetable oil consumption, with no language restriction. The search terms are described in Supplemental Text 1. This was supplemented with a review of the reference lists of eligible reviews and meta-analyses.

Studies were included if they met the following criteria: *1*) systematic reviews or meta-analyses of studies in adults that *2*) investigated the association of different vegetable oils (e.g., soybean oil, palm oil, peanut oil, and sunflower oil) on *3*) health outcomes, (e.g., lipid parameters, weight). Studies were excluded if they were *1*) primary studies, *2*) vegetable oils that are used in the form of dietary supplements, or *3*) the individual sterol components of the vegetable oils (e.g., β-sitosterol, campesterol, and brassicasterol). Publications that reported only on the exposure to plasma concentrations or biomarkers without the dietary intake of vegetable oils were further excluded.

### Data extraction

Two authors independently extracted the data. When the study included a meta-analysis, the effects of each comparison on health outcomes, along with their 95% confidence intervals (CIs) and quality scores, were also extracted. All extracted data were double checked and verified by the senior author (SWHL), and any disagreement was resolved through consensus. If multiple published meta-analyses were identified on the same association, the primary studies from each meta-analysis were extracted for each exposure to avoid the inclusion of duplicate studies.

### Assessment of methodological quality

The assessment of the methodological quality of each included published meta-analysis was conducted independently by 2 reviewers using the validated AMSTAR-2 (A measurement tool to assess systematic review) tool [9]. Subsequently, the assessments were examined by another 2 reviewers. In cases of disagreement and failed consensus, further consultation was obtained from a third reviewer (SWHL).

### Data analysis

#### Assessment of summary effects

All outcomes were synthesized and narratively described, with the findings presented in a tabular format. The review characteristics and findings were presented in the summary table. The meta-analysis and corresponding 95% CI were recalculated using the DerSimonian and Laird random effects model for each outcome. This was performed using summary effects of the primary studies that compared 2 different oils reported in the published meta-analyses. If 2 or more reviews assessed the same outcome and consumption of edible oil, data from the latest study were included. However, both sets of findings were compared. The quality of evidence provided in the meta-analyses was evaluated using the GRADE (Grading of Recommendations, Assessment, Development, and Evaluations) criteria. The evidence was rated from very low, low, moderate, and high certainty in the evidence based on the 5 recommended domains [10].

Heterogeneity was examined through Cochran’s Q test and *I*^2^ statistic with the identification of heterogeneity based on a *P* value of <0.1 or *I*^2^ ≥50%. Funnel plots were visually inspected to assess publication bias and small study effects. STATA version 16.0 (StataCorp LLC) was used for all analyses.

## Results

### Literature search

**FIGURE 1:**
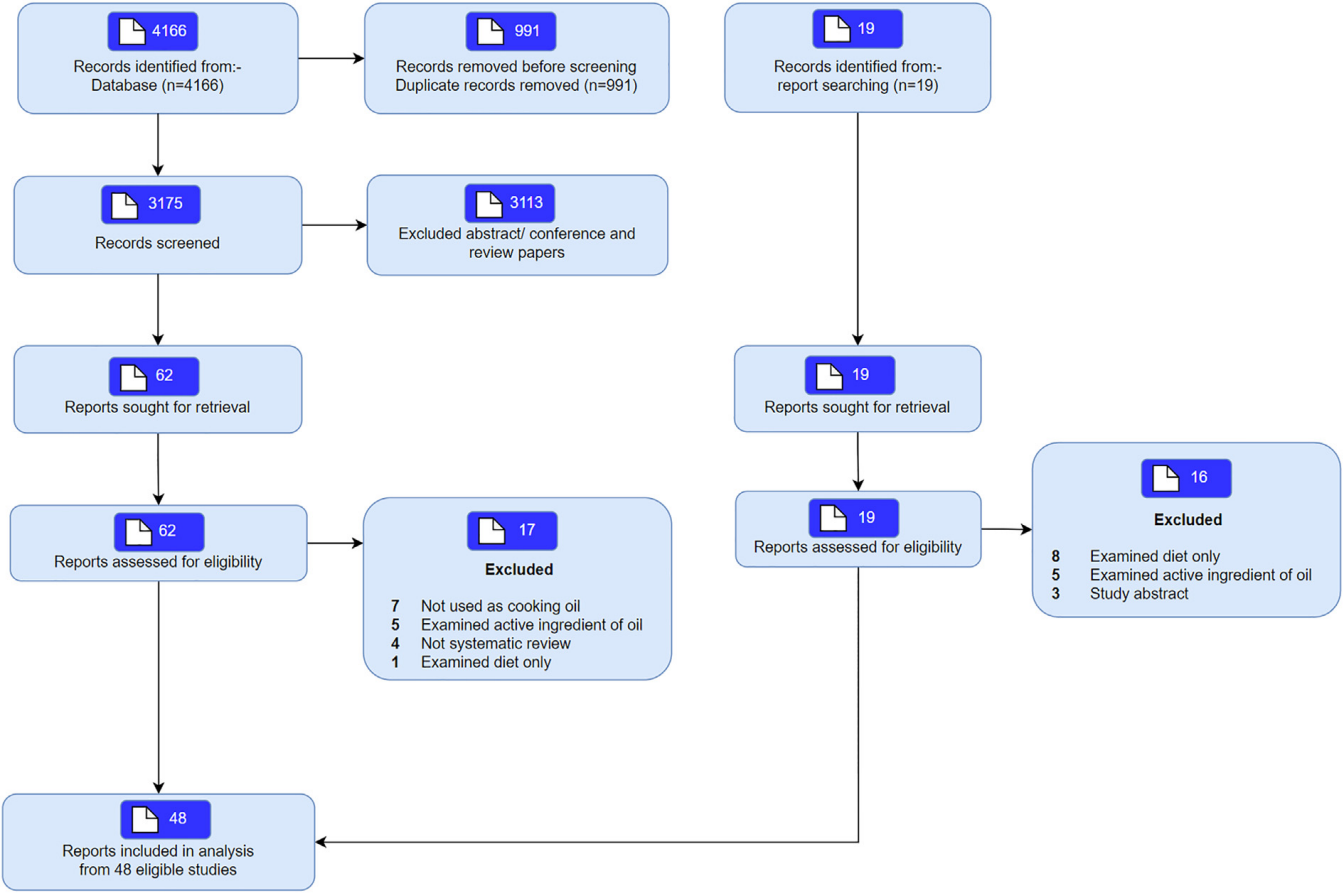
PRISMA flow chart showing the selection of studies. PRISMA, preferred reporting items for systematic reviews and meta-analyses.

The literature search identified 4166 articles, of which 3175 articles were screened. Eighty articles were reviewed, with 48 articles included in the final umbrella review. Reasons for exclusion included conference abstracts (*n* = 3), noncooking oils (*n* = 7), examined effects of diet (*n* = 9), not systematic review (*n* = 4), and they examined active ingredients of oils (*n* = 10) (Figure 1 and Supplemental Text 2).

Studies including olive oil (*n* = 9) [[11], [12], [13], [14], [15], [16], [17], [18], [19]], coconut oil (*n* = 9) [[20], [21], [22], [23], [24], [25], [26], [27], [28]], flaxseed oil (*n* = 8) [[29], [30], [31], [32], [33], [34], [35], [36]], palm oil (*n* = 7) [[37], [38], [39], [40], [41], [42], [43]], canola oil (*n* = 6) [[44], [45], [46], [47], [48], [49]], sesame oil (*n* = 3) [[50], [51], [52]], rice bran oil (*n* = 2) [53,54], virgin olive oil (*n* = 2) [55,56], palm olein (*n* = 1) [57] and peanut oil (*n* = 1) [58] consumption and their health outcomes were examined (Table 1 [[11], [12], [13], [14], [15], [16], [17], [18], [19], [20], [21], [22], [23], [24], [25], [26], [27], [28], [29], [30], [31], [32], [33], [34], [35], [36], [37], [38], [39], [40], [41], [42], [43], [44], [45], [46], [47], [48], [49], [50], [51], [52], [53], [54], [55], [56], [57], [58]]). Of these, 39 studies included meta-analyses that reported 206 summary odds ratio, risk ratio (RR), or mean differences for respective health outcomes. These meta-analyses investigated the effects of the consumption of edible oils on the following measures: lipid parameters (*n* = 115), anthropometric measurements (*n* = 30), blood pressure outcomes (*n* = 16), glycemic outcomes (*n* = 30), inflammatory markers (*n* = 2), CVD risk (*n* = 10) and cancer risk (*n* = 3). These associations were evaluated in a heterogeneous population, which included healthy adults, those with hyperlipidemia, diabetes, chronic heart diseases, or overweight individuals. Visual inspection of the funnel plot did not identify the presence of publication bias. Strength of evidence using GRADE found that the associations were supported by very low- (*n* = 139; 67.5%) and low- (*n* = 45; 21.8%) strength of evidence. The remaining were supported by moderate- (*n* = 20; 9.7%) and high- (*n* = 2; 1.0%) strength of evidence.

**TABLE 1:** Characteristics of included studies in the current review

Source	Study type	Oil type	Comparator	Number of studies included in the review	Health outcome
Al-Ghamdi 2018 [17]	Systematic review and meta-analysis	Mediterranean diet rich in olive oil	Mixed nuts or low-fat diet	4	Cardiovascular disease risk: cardiovascular events, cardiovascular death, all-cause death
Amiri 2020 [47]	Systematic review and meta-analysis	Canola oil	Olive oil, palm oil, sunflower oil, soybean oil, safflower oil, coconut oil, rice bran oil, flaxseed oil	44	Lipid parameters: TC, TG, LDL cholesterol, HDL, VLDL (mmol/L), LDL/HDL ratio, TC/HDL ratio, TGs in HDL, TGs in LDL, TGs in VLDL, apolipoprotein A-1, apolipoprotein B, apolipoprotein B to apolipoprotein A-1 ratio, lipoprotein Blood glucose control markers: fasting blood sugar, insulin, HOMA-IR Blood pressure: systolic blood pressure, diastolic blood pressure Inflammatory marker: C-reactive protein
Atefi 2022 [52]	Systematic review and meta-analysis	Sesame oil	Soybean oil, coconut oil, sunflower oil, olive oil,	12	Anthropometric indices: weight, BMI Blood pressure: systolic blood pressure, diastolic blood pressure Blood glucose control markers: fasting blood sugar, insulin Inflammatory markers: malondialdehyde
Azad 2020 [58]	Systematic review and meta-analysis	Peanut oil	Canola oil, sesame oil, safflower oil, sunflower oil, olive oil	11	Lipid parameters: TC, TG, LDL cholesterol, HDL Anthropometric indices: weight, waist circumference, BMI Blood pressure: systolic blood pressure, diastolic blood pressure Blood glucose control markers: fasting blood sugar, insulin
Basch 2007 [34]	Systematic review	Flaxseed oil	Details were not reported	18	Lipid parameters Cancer risk Blood glucose control
Cardoso 2018 [51]	Systematic review	Sesame oil	Details were not reported	7	Lipid parameters: TC, LDL cholesterol, HDL cholesterol, TGs Blood pressure: systolic blood pressure, diastolic blood pressure
Cortez-Ribeiro 2023 [19]	Systematic review	Olive oil	Details were not reported	9	Maternal outcome: gestational diabetes mellitus prevalence, preeclampsia, cardiovascular disease risk, hypertension Fetal outcomes: small or large for gestational age, prematurity
Dhanasekara 2022 [26]	Systematic review and meta-analysis	Coconut oil	Soybean oil, olive oil, palm oil, sea buckthorn berry oil, butter, peanut oil, sesame oil	18	Blood glucose control markers: AUC of insulin, AUC glucose, HOMA-IR, HOMA-β, fasting blood sugar, insulin
Dong 2017 [38]	Systematic review and meta-analysis	Palm oil (red palm oil)	Groundnut oil, sunflower oil	9	Anthropometric indices: body weight
Duarte 2022 [25]	Systematic review and meta-analysis	Coconut oil	Olive oil Soybean oil, sunflower oil, chia oil, safflower oil, palm oil, butter	17	Anthropometric indices: body weight, waist circumference Lipid parameters: LDL cholesterol, HDL cholesterol, TG, TC/HDL cholesterol ratio Blood glucose control markers: fasting blood sugar Inflammatory markers: ultra-sensitive C-reactive protein
Eyres 2016 [23]	Systematic review	Coconut oil	Butter diet, soybean oil, palm olein or corn oil, palm oil, extra virgin olive oil, habitual diet	21	Lipid parameters: TC, LDL cholesterol, HDL cholesterol, TGs
Fattore 2014 [37]	Systematic review and meta-analysis	Palm oil	Diet-enriched fat with peanut oil, sunflower oil, safflower oil, stearic acid, oleic acid, soybean oil, coconut oil, elaidic acid, myristic acid, peanut oil, canola oil	53	Lipid parameters: VLDL, LDL/HDL ratio
George 2019 [55]	Systematic review and meta-analysis	High polyphenol diet	Low polyphenol diet	15	Lipid parameters: TC, LDL cholesterol, oxidized LDL, HDL Blood pressure: systolic blood pressure, diastolic blood pressure Oxidative stress markers: malondialdehyde concentrations, oxidized LDL, total antioxidant capacity, glutathione peroxidase concentrations
Ghobadi 2019a [46]	Systematic review and meta-analysis	Canola oil	Sunflower oil, rice bran oil, olive oil, safflower oil, corn oil, soybean oil, palm oil, coconut oil	27	Lipid parameters: TC, TG, LDL, HDL, LDL/HDL, TC/HDL, apolipoprotein A-1, apolipoprotein B
Ghobadi 2019b [11]	Systematic review and meta-analysis	Olive oil	Soybean oil, palm oil, corn oil, rapeseed oil, safflower oil, sesame oil, flaxseed oil, rice bran oil	27	Lipid parameters: TC, TG, LDL cholesterol, HDL
Gouveia 2016 [50]	Systematic review	Sesame oil	NA	7	Oxidative stress markers
Harland 2009 [44]	Systematic review and meta-analysis	Canola oil	Mixed fat, customary, control, saturated fat diet, palm control, baseline diet	10	Lipid parameters: TC, LDL
Hisham 2020 [43]	Systematic review and meta-analysis	Palm oil	Coconut oil, sunflower oil	21	Lipid parameters: apolipoprotein A-1, apolipoprotein B
Hohmann 2015 [56]	Systematic review and meta-analysis	Virgin olive oil	Refined olive oil without/with reduced phenolic content	8	Blood pressure: systolic blood pressure, diastolic blood pressure Oxidative stress markers: oxidized LDL, malondialdehyde Lipid parameters: TC, HDL, LDL
Ismail 2018 [40]	Systematic review	Palm oil	Soybean oil	4	Cardiovascular disease risk: coronary artery disease, stroke
Jayawardema 2020 [24]	Systematic review and meta-analysis	Coconut oil	Soybean oil, safflower oil, corn oil, palm oil, butter, olive oil, peanut oil, canola oil	20	Lipid parameters: TC, TG, LDL cholesterol, HDL, VLDL, LDL/HDL ratio, TC/HDL ratio, TG/HDL ratio, apolipoprotein A-1, apolipoprotein B Blood glucose control markers: HbA1c, fasting blood sugar, BMI
Jolfaie 2016 [53]	Systematic review and meta-analysis	Rice bran oil	Peanut oil, olive oil, corn oil, canola oil	11	Lipid parameters: TC, TG, LDL cholesterol, HDL, VLDL, LDL/HDL ratio, TC/HDL ratio, apolipoprotein A-1, apolipoprotein B, lipoprotein
Khalesi 2015 [32]	Systematic review and meta-analysis	Flaxseed oil	Sunflower oil, soybean oil, maltodextrin, wheat germ, wheat barn, rice flour, wheat, hempseed oil	11	Blood pressure: systolic blood pressure, diastolic blood pressure
Lin 2013 [45]	Systematic review	Canola oil	Control/baseline diet: palm oil, corn oil, milk fat, flaxseed oil, coconut oil, olive oil, sunflower oil butter, margarine, cream, high-fat cheese	31	Lipid parameters: TC, LDL cholesterol, HDL cholesterol, TGs Inflammatory markers: C-reactive protein, IL-6, sVCAM-1, sICAM-1, soluble E-selectin, P-selectin, L-selectin, TNF-α
Ma 2016 [20]	Systematic review	Coconut oil	Details were not reported	Details were not reported	Lipid parameters: LDL, HDL, apolipoprotein A-1 Blood pressure Blood glucose control
Mahmudiono 2022 [36]	Systematic review and meta-analysis	Flaxseed oil	Sunflower oil, soybean oil, safflower oil, corn oil	5	Blood pressure: systolic blood pressure, diastolic blood pressure
Martínez-González 2014 [18]	Systematic review and meta-analysis	Olive oil	Different amount of olive oil/control diet	9	Cardiovascular disease risk: risk of developing chronic heart disease, risk of developing chronic heart disease, risk of developing stroke
Mohammadi-Sartang 2017 [35]	Systematic review and meta-analysis	Flaxseed oil	Sunflower oil, soybean oil, corn oil	45	Anthropometric indices: body weight, BMI, waist circumference
Neelathakan 2020 [21]	Systematic review and meta-analysis	Coconut oil	Soybean oil, safflower oil, peanut oil, olive oil, corn oil	16	Inflammatory marker: C-reactive protein Anthropometric indices: body weight, waist circumference, body fat
Pan 2009 [33]	Systematic review and meta-analysis	Flaxseed oil	sunflower seed, sunflower oil, wheat bran, wheat flour, wheat germ, manioc flour, safflower oil, olive oil, hypolipidemic, canola oil, wheat, psyllium	28	Lipid parameters: TC, LDL cholesterol, HDL cholesterol, TGs
Pourrajab 2021 [54]	Systematic review and meta-analysis	Rice bran oil	Peanut oil, olive oil, virgin olive oil, corn oil, canola oil, palm oil, butter, soybean oil, liquid lard, standard spread, sunflower oil	8	Lipid parameters: TC, LDL cholesterol, HDL cholesterol, TGs
Pourrajab 2022 [49]	Systematic review and meta-analysis	Canola oil	Olive oil	13	Lipid parameters: HDL cholesterol, LDL cholesterol, TC, TG, TC/HDL cholesterol ratio, LDL cholesterol /HDL cholesterol ratio, VLDL cholesterol
Psaltopoulou 2011 [16]	Systematic review and meta-analysis	Diet supplemented with olive oil	Details were not reported	38	Cancer risk: risk of developing breast cancer, risk of developing digestive cancer, risk of developing other cancer
Raeisi-Dehkordi 2019 [48]	Systematic review and meta-analysis	Canola oil	Rapeseed oil, sunflower oil, flaxseed oil	25	Anthropometric indices: body weight, BMI, waist circumference, body fat waist hip ratio, android to gynoid fat ratio, hip circumference, lean mass
Ren 2016 [29]	Systematic review and meta-analysis	Flaxseed oil	Soybean oil, olive oil, safflower oil	20	Inflammatory marker: C-reactive protein
Schwingshackl 2014 [13]	Systematic review and meta-analysis	Olive oil	Other monosaturated fatty acid	32	Cardiovascular disease risk: all-cause mortality, cardiovascular mortality, combined cardiovascular events, stroke, coronary artery disease
Schwingshack 2015 [12]	Systematic review and meta-analysis	Olive oil: Diet supplemented with olive oil	Low-fat diet, healthy diet, flaxseed oil, palm oil, coconut oil, sunflower oil	28	Inflammatory markers: C-reactive protein, IL-6, TNF-α, sP-selectin, intracellular adhesion molecule-1, vascular cell adhesion molecule-1-1
Schwingshack 2017 [14]	Systematic review and meta-analysis	Olive oil	Sunflower oil, cod liver oil, fish oil, krill oil	29	Blood glucose control markers: fasting blood sugar, HbA1c, risk of developing type 2 diabetes
Sekhar 2022 [27]	Systematic review	Coconut oil	Soybean oil, safflower oil, palm oil, corn oil, extra virgin olive oil, sunflower oil, butter,	5	Lipid parameters: TC, TG, LDL cholesterol, HDL
Sun 2015 [41]	Systematic review and meta-analysis	Palm oil	Peanut oil, olive oil, canola oil, soybean oil, sunflower oil, corn oil	30	Lipid parameters: TC, TG, LDL cholesterol, HDL
Swarnamali 2023 [28]	Systematic review and meta-analysis	Coconut oil	Soybean oil, corn oil, safflower oil, peanut oil, chia oil, sunflower oil	9	Anthropometric indices: body weight, body fat, waist circumference, waist hip ratio, BMI, body weight
Teng 2020 [22]	Systematic review and meta-analysis	Coconut oil	Soybean oil, diet, safflower oil, olive oil, canola oil, palm oil, peanut oil	18	Lipid parameters: LDL cholesterol, HDL cholesterol, TG
Ursoniu 2016 [30]	Systematic review and meta-analysis	Flaxseed oil	Sunflower oil, safflower oil, olive oil	15	Blood pressure: systolic blood pressure, diastolic blood pressure
Ursoniu 2019 [31]	Systematic review and meta-analysis	Flaxseed oil	Details were not reported	7	Inflammatory marker: plasma C-reactive protein concentrations
Voon 2019 [57]	Systematic review and meta-analysis	Palm olein	Canola oil, coconut oil, soybean oil, sunflower oil, peanut oil, olive oil	9	Lipid parameters: TC/HDL ratio
Wang 2019 [39]	Systematic review and meta-analysis	Palm oil	Olive oil, oleic acid, canola oil, sunflower oil, peanut oil, soybean oil, soya oil, corn oil	11	Lipid parameters: TC, LDL cholesterol, HDL cholesterol, TG
Zamora 2018 [15]	Systematic review and meta-analysis	Diet supplemented with olive oil	Low-fat diet, healthy diet, sunflower oil	11	Anthropometric indices: body weight, BMI, waist circumference
Zulkiply 2018 [42]	Systematic review and meta-analysis	Palm oil	Partially hydrogenated soybean oil diets	8	Blood glucose control markers: fasting plasma glucose, fasting insulin

Abbreviations: AUC, area under the curve; BMI, body mass index; HbA1c, hemoglobin A1c; HDL, high-density lipoprotein; HOMA-IR, homeostasis model assessment of insulin resistance; IL, interleukin; LDL, low-density lipoprotein; TC, total cholesterol; TG, triglyceride; TNF, tumor necrosis factor; VLDL, very low-density lipoprotein; sICAM-1, soluble intercellular adhesion molecule-1; sVCAM-1, vascular cell adhesion molecule-1

### Methodological quality

AMSTAR-2 tool analysis found that only 1 included review was evaluated as high in confidence level. Seven (14.6%) reviews were moderate confidence, 30 (62.5%) were low confidence, and 10 (20.8%) were rated as critically low confidence (Supplemental Figures 1 and 2). These reviews were rated low or critically low confidence as they had the following critical flaw(s) with 35 reviews lacking a list of excluded studies with justification for their exclusion; 17 reviews did not address the risk of bias in the interpretation of the results, and 14 reviews did not mention the establishment of the review methods before the reviews were conducted (Supplemental Figures 1 and 2).

### Summary of findings

The main results, together with the effect sizes for each of the reported outcomes, were summarized in Table 2. Based on the random effects model, with 206 analyses performed, 47 (22.8%) were found statistically significant. These results were supported mainly by a very low strength of evidence (*n* = 26; 55.3%), followed by low strength of evidence (*n* = 11; 23.4%), moderate strength of evidence (*n* = 8; 17.0%) and high strength of evidence (*n* = 2; 4.3%), and were primarily related to lipid outcomes and anthropometric measurements (Supplemental Tables 1–10).

**TABLE 2:**
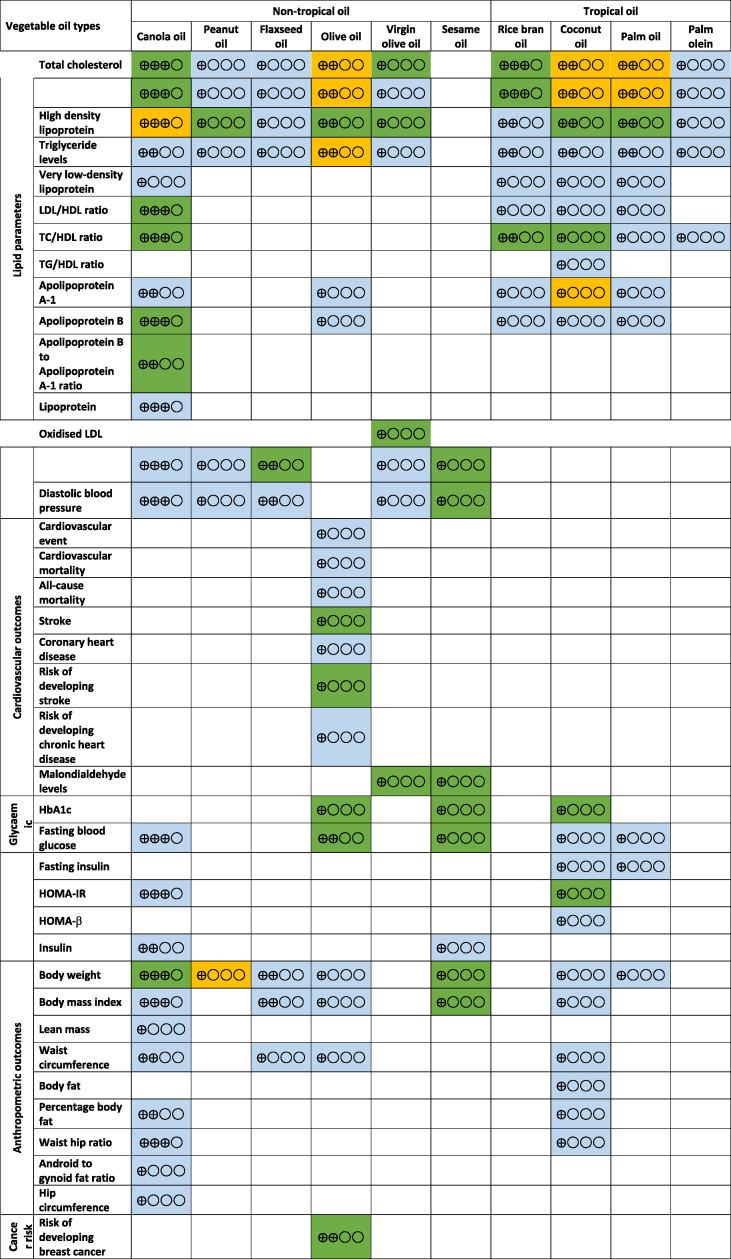

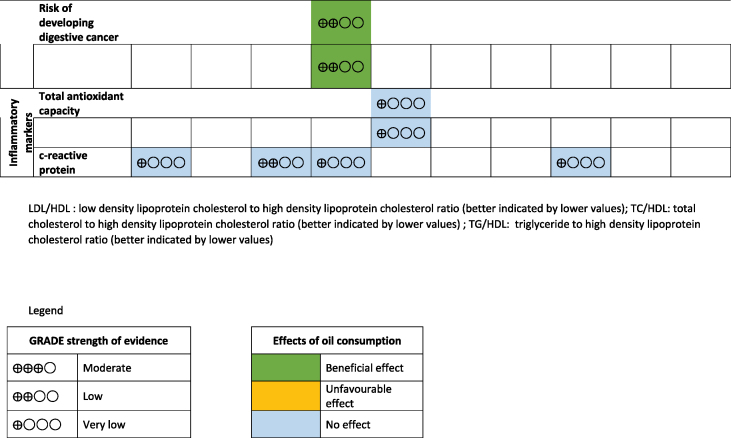
Study summary of the included reviews

Overall, some beneficial effects on lipid profiles were reported with the consumption of canola, virgin olive, and rice bran oil, whereas the consumption of sesame and flaxseed oil was found to improve blood pressure. Meanwhile, the consumption of both sesame and olive oils was reported to improve glycaemic profiles, whereas sesame oil consumption could reduce body weight and BMI (in kg/m^2^), and the consumption of olive oil reduced some cancer risk. The full description of these effects is described in detail below.

### Lipid parameters

Lipid parameters were the most commonly reported health outcomes in all studies. A total of 24 studies reported the effects of vegetable oils on lipid parameters: coconut oil (*n* = 5), [20,[22], [23], [24], [25]], canola oil (*n* = 5) [[44], [45], [46], [47],^49^], palm oil (*n* = 5) [37,39,41,43,57], olive oil (*n* = 3) [11,55,56], rice bran oil (*n* = 2) [53,54], flaxseed oil (*n* = 2) [33,34], peanut oil (*n* = 1)[58] and sesame oil (*n* = 1) [51]. The degree of effects on lipid parameters, namely serum total cholesterol (TC), serum triglyceride (TG), serum LDL cholesterol, serum VLDL, serum HDL cholesterol, TC: HDL ratio, LDL: HDL ratio, TG: HDL ratio, and apolipoproteins varied among the vegetable oils. Canola oil, virgin olive oil, and rice bran oil were found to significantly reduce serum TC between 0.86 mmol/L and 0.11 mmol/L compared to other vegetable oils (Table 2). Conversely, the use of coconut oil, olive oil, and palm oil increased serum TC significantly between 0.19 mmol/L and 0.40 mmol/L.

The consumption of canola oil [47,49] and rice bran oil [54] was found to reduce LDL concentrations, whereas coconut oil, palm oil, and olive oil increased serum LDL. On the contrary, coconut oil [22,24,25], palm oil [39], peanut oil [58], virgin olive oil [55], and olive oil [11,55] were all found to increase serum HDL significantly. Consumption of canola oil [47], coconut oil [24], palm oil [37], or rice bran oil [53] did not significantly affect serum VLDL concentrations. Only olive oil [11] increased serum TG concentrations, whereas other plant oils showed no significant effect.

In terms of cholesterol ratio, the effects of vegetable oils on LDL: HDL, TC: HDL, and TG: HDL were heterogeneous. However, canola oil was found to reduce apolipoprotein B concentrations [47], whereas coconut oil increased apolipoprotein A1 concentrations [24]. Olive oil [11], rice bran oil [53], and palm oil [43] were found to have no significant effects on apolipoprotein concentrations.

### Blood pressure, inflammatory markers, and cardiovascular outcomes

Studies reported the pooled effect of canola oil [47], flaxseed oil, olive oil [55,56], sesame oil [52], and peanut oil [58] on blood pressure (systolic and diastolic). Only flaxseed oil and sesame oil were found to reduce blood pressure [30,32,52]. No changes in C-reactive protein concentrations were reported with canola oil [47], flaxseed oil [29], olive oil [12], and coconut oil [21]. Besides that, olive oil was found to have no significant effect on the risk for cardiovascular events (RR: 0.97; 95% CI: 0.67, 1.39), cardiovascular deaths (RR: 1.07; 95% CI: 0.77, 1.48), and all-cause deaths (RR: 0.99; 95% CI: 0.85, 1.15) [17]. Very weak evidence also suggests that olive oil could reduce the risk of developing stroke [18].

### Glycaemic outcomes

Reports on the effect of vegetable oil consumption on blood sugar controls were scarce. Hemoglobin A1c was found to decrease significantly with coconut oil [24], olive oil [14], and sesame oil [52], whereas fasting blood glucose concentrations were reduced with olive oil [14] and sesame oil [52]. No significant effects were found on fasting blood glucose concentrations or HOMA-IR with canola oil [47], peanut oil [58], coconut oil [24], and palm oil [42].

### Anthropometric indices

The use of canola oil and sesame oil was reported to reduce body weight between 0.35 kg and 0.30 kg; however, peanut oil consumption has been shown to increase weight (0.90 kg; 95% CI: 0.40, 1.40). No significant effects were found on waist circumference, body fat, waist: hip ratio, android: gynoid fat ratio, hip circumference, or lean mass for all other vegetable oils.

### Cancer risk

Only 1 study investigated the effect of olive oil consumption on the risk of developing cancers [16]. The report indicated that olive oil consumption was associated with lower odds of developing cancer, including breast cancer and digestive cancer [16].

## Discussion

To the best of our knowledge, this umbrella review represents the first effort to synthesize the evidence from existing systematic reviews and meta-analyses on the effects of vegetable oils on health outcomes. The present review supports the growing body of evidence suggesting vegetable oils rich in MUFAs and PUFAs, such as canola oil and rice bran oil, have desirable effects in reducing TC and LDL concentrations. MUFAs and PUFAs are anti-atherogenic and anti-inflammatory and demonstrate beneficial effects on cholesterol concentrations, consistent with epidemiological and clinical data [59]. Notably, the current review showed that only virgin olive oil [11] enriched with higher concentrations of polyphenols are commonly associated with additional health benefits, including antioxidant effects, anti-atherosclerotic potential, and anti-inflammatory properties that lower lipid parameters.

In contrast, we found that oils relatively rich in saturated fats, such as coconut and palm oil, tend to increase TC and LDL. On the contrary, coconut oil has gained popularity in recent years for its potential health benefits attributed to the presence of medium-chain TGs that are absorbed intact and directly in the liver [60]. However, our present review found limited evidence supporting these claims. On the contrary, it has been reported that palm oil can adversely affect blood lipid profiles due to its higher saturated fat content when compared to other vegetable oils, which contain lower concentrations of saturated fats [42]. Such effects may be associated with CVD risks, but this needs to be taken in light of the beneficial effects of increasing HDL concentrations.

Palm oil is a versatile product that can be fractionated into palm olein, palm stearin, and palm mid-fractions. These palm oil fractions have very different physicochemical and nutritional properties as well as functions. Therefore, it is imperative for this study to differentiate between these products and avoid categorizing them under the common term “palm oil.” Our review found that when focusing on palm olein, the liquid fraction of palm oil commonly used in cooking in many Southeast Asian countries, it has minimal impact on lipid parameters when compared to other vegetable oils rich in unsaturated fatty acids, such as olive oil, canola oil, soybean oil, and high-oleic sunflower oil. This observation may be attributed to the positional distribution of fatty acids on the TG backbone present in palm olein [57,61]. Additionally, palm olein exhibits a higher degree of unsaturation, with elevated concentrations of oleic acid (39–45%) and LA (10–13%) [61]. Similar to other vegetable oils rich in unsaturated fatty acids, the stereospecific number-2 position of the TG backbone in palm olein predominantly contains MUFA and PUFA, which are known to influence lipid profiles, a topic that has been discussed [62,63].

There is now a growing interest in the consumption of virgin oils as functional foods compared to the refined form of the oils [64]. This interest stems from the refining processes involved, which may reduce the amount of antioxidants and polyphenols in the oils, compounds known for their health-protective effects [20]. For instance, virgin olive oil, which contains high concentrations of polyphenols, can reduce oxidative stress that is often associated with chronic diseases [55]. Although our current review aimed to explore the benefits of different oils and their extraction methods, we only identified 2 studies that have examined this aspect, which reported the health effects of virgin olive oil consumption compared to olive oil [55,56]. Therefore, future research should delve deeper into this topic and address any existing controversies.

The health benefits of various vegetable oils were also reflected in other outcomes to an extent, including blood pressure, hemoglobin A1c, weight and BMI. Of note, the consumption of flaxseed oil demonstrated a slight reduction of ≤4.10 mmHg in diastolic blood pressure, which is nearing the clinical relevance associated with lower disease risks [65]. The distinctive feature of flaxseed oil is the amount (>50%) of (n–3) ALA that can be converted to EPA and DHA [1]. Along with EPA and DHA, ALA was also suggested to have cardioprotective potential, thereby improving blood pressure [32].

Our current review complements the recent review by Teasdale et al. [66], which reported the profile of nutrients and bioactive contents of edible oils and fats to better inform dietary recommendations, albeit with some differences. Unlike our review, they attempted to delineate the impact of various bioactive contents in each oil. For instance, the review found that most studies to date have reported some beneficial effects on the cardiovascular effects of these edible oils. These beneficial effects were generally attributed to the use of biophenols and flavonoids, which can be found in oils such as extra virgin olive oils, coconut oils, hemp seed, and avocado oil. Emerging evidence also suggests some beneficial effects with the use of tocopherols and squalene, which are often rich in oils such as palm oil, soybean oil, canola oil, and sunflower oil.

### Strengths and limitations

Our study demonstrated several strengths. The study offers a comprehensive systematic overview of the evidence from all published systematic reviews and meta-analyses on the health benefits of various vegetable oils. The methodological quality of reviews was assessed, and the quality of evidence was graded using validated tools. However, this study has certain limitations which cannot be neglected. First, the primary studies included in each meta-analysis were not assessed directly. Therefore, the results could have been influenced by primary studies not included in the published meta-analyses or additional studies published after the reviews. Recalculation for 54 of the 206 meta-analyses was impossible as no estimates were reported. We also could not determine the populations examined for meal compositions, including the types of fatty acids (SFA, MUFA, and PUFA) intake and their quantities, which are important for interpreting results. For example, studies that used standardized feeding methodology might have provided a more accurate representation of the effects of vegetable oils on lipid and glycemic outcomes rather than cross-sectional studies that confounding variables might influence.

Additionally, the populations examined in all studies were heterogeneous and might have included those with hyperlipidemia; therefore, they might not reflect a true direct comparison of the effects. As such, to derive recommendations, further investigations are warranted. Our analysis included the largest number of primary studies for each outcome or most recent publication and oil type. Therefore, we might not have chosen meta-analyses with the highest quality of evidence. Nevertheless, most updated reviews often included the same primary studies as prior reviews. Finally, due to the limited number of primary studies, most of the outcomes reported in our umbrella review were only of low-quality evidence. As such, including a meta-analysis with even fewer primary studies would not change the quality of evidence compared to those in our umbrella review.

### Implications for practice

This review has identified several significant potential implications for the public, clinicians, and policymakers. Firstly, limited evidence shows that consumption of vegetable oils rich in MUFA and PUFA instead of SFAs can reduce coronary events, including mortality, despite various mechanisms and evidence supporting MUFA- and PUFA-rich vegetable oils benefits in lowering lipid concentrations in adults with or without comorbidities [47,53]. Secondly, this review also noted that the different types of vegetable oils offer different health benefits due to their unique fatty acid profiles. It is crucial to emphasize that the reported health benefits of these vegetable oils consumed are based on the amounts recommended in dietary guidelines and not by overconsumption/increasing the total amounts of calories consumed daily, as this may lead to weight gain and possibly obesity.

Finally, it is necessary to implement research investigating the effects of the long-term safety and efficacy of various vegetable oils, given that most of the reviews included in this study only examined RCTs, which are often conducted over a short period of ≤6 mo. Studies should also explore the question of internal consistencies, such as comparing virgin coconut oil with coconut oil and the influence of mediating factors such as body fats.

In conclusion, numerous studies have examined the effects of various vegetable oils on health outcomes. This review suggests that different vegetable oils offer different health benefits, which provide potential primary preventive effects of diseases. Given the challenges in evaluating the impacts of various vegetable oils independent of other dietary practices and the trial durations studied, upcoming research should prioritize obtaining comprehensive dietary data and concentrate on long-term clinical outcomes such as cardiovascular events and mortality.

## Acknowledgments

We thank Muhammad Iqbal Abu Bakar and Avonnie Chee Yuen Chi for their assistance throughout the study.

## Author contributions

The authors’ responsibilities were as follows – PTV, CMN, YTN, YJW, SYY, SLL, XSY, SWHL: designed, conducted, analyzed the systematic literature search, and provided inputs to the writing of the manuscript; PTV, YTN, SYY, XSY, SWHL: cross-checked the databases for any duplication of articles selected; PTV, SWHL: was responsible for the final content check; SWHL: double checked, verified, and consulted the whole working process; and all authors: read and approved the final manuscript.

## Conflict of interest

The authors report no conflicts of interest.

## Funding

The authors reported no funding received for this study.
