# Supplementary material for: Health Effects of Various Edible Vegetable Oil: An Umbrella Review
Source: Adv Nutr. 2024 Jul 23;15(9):100276. doi: 10.1016/j.advnut.2024.100276 (PMC11374968; doi:10.1016/j.advnut.2024.100276)
Supplement: Multimedia component 1 [file mmc1.docx]

**Health effects of various edible vegetable oil: An umbrella review**

Voon et al

**Supplementary Text S1**

A comprehensive search was performed to identify for both published and unpublished information. A sample of search terms used is shown below and adapted to each individual database, including PubMed, EMBASE, PsychINFO, Database of abstracts of review of effectiveness (DARE), HTA database and report, NHS EED, HealthSTAR, BIOSIS, Science Citation Index, Cochrane central register of controlled trials (CENTRAL), CINAHL Plus and AMED.

List of keywords used in PubMed

1. Fatty acids, Omega-6.ti,ab
2. Fatty acids, Omega-3.ti,ab
3. Fatty acids unsaturated.ti,ab
4. Fatty acids, monounsaturated.ti,ab
5. Trans fatty acids.ti,ab
6. monounsaturated.ti,ab
7. mono-unsaturated.ti,ab
8. unsaturated.ti,ab
9. polyunsaturated.ti,ab
10. saturated.ti,ab
11. trans-unsaturated.ti,ab
12. trans-fatty.ti,ab
13. trans fatty.ti,ab
14. trans unsaturated.ti,ab
15. trans fat.ti,ab
16. omega-6.ti,ab
17. omega-3.ti,ab
18. palmitic.ti,ab
19. palmitate.ti,ab
20. stearic.ti,ab
21. stearate.ti,ab
22. myristic.ti,ab
23. myristate.ti,ab
24. lauric.ti,ab
25. laurate.ti,ab
26. SFA.ti,ab
27. oleic.ti,ab
28. oleate.ti,ab
29. palmitoleic.ti,ab
30. palmitoleate.ti,ab
31. MUFA.ti,ab
32. linoleic.ti,ab
33. lonoleate.ti,ab
34. octadecadienoic acid.ti,ab
35. PUFA.ti,ab
36. Vaccenic acid.ti,ab
37. vaccenate.ti,ab
38. conjugated linoleic.ti,ab
39. CLA.ti,ab
40. TFA.ti,ab
41. Coconut oil.ti,ab
42. Seed oil.ti,ab
43. Safflower oil.ti,ab
44. Sunflower oil.ti,ab
45. Hempseed oil.ti,ab
46. Canola oil.ti,ab
47. Sesame oil.ti,ab
48. Soybean oil.ti,ab
49. Soyabean oil.ti,ab
50. Rapeseed oil.ti,ab
51. Corn oil.ti,ab
52. Olive oil.ti,ab
53. Nut oil.ti,ab
54. Linseed oil.ti,ab
55. Flaxseed oil.ti,ab
56. Grapeseed oil.ti,ab
57. Peanut oil.ti,ab
58. Groundnut oil.ti,ab
59. Avocado oil.ti,ab
60. Palm oil.ti,ab
61. Vegetable oil.ti,ab
62. margerine.ti,ab
63. hydrogenated oil.ti,ab
64. #1 to #63/OR
65. Health outcomes.ti,ab
66. Blood pressure.ti,ab
67. health.ti,ab
68. glucose.ti,ab
69. hba1c.ti,ab
70. glycated haemoglobin.ti,ab
71. fasting plasma glucose.ti,ab
72. fasting glucose.ti,ab
73. systolic blood pressure.ti,ab
74. diastolic blood pressure.ti,ab
75. lipid.ti,ab
76. triglyceride.ti,ab
77. total cholesterol.ti,ab
78. low density lipoprotein.ti,ab
79. cholesterol.ti,ab
80. high density lipoprotein.ti,ab
81. quality of life.ti,ab
82. body weight.ti,ab
83. body mass index.ti,ab
84. BMI.ti,ab
85. cardiovascular.ti,ab
86. endocrine.ti,ab
87. cancer.ti,ab
88. #65 to #87/OR
89. Systematic review
90. Meta analysis
91. 89 OR 90
92. #64 AND #88
93. #92 AND #91

**Supplementary Text S2**

**List of excluded studies**

**Not systematic review**

- Anton, S. D., Heekin, K., Simkins, C., & Acosta, A. (2013). Differential effects of adulterated versus unadulterated forms of linoleic acid on cardiovascular health. Journal of integrative medicine, 11(1), 2–10.
- Covas M. I. (2007). Olive oil and the cardiovascular system. Pharmacological research, 55(3), 175–186.
- Shayan, M., Kamalian, S., Sahebkar, A., & Tayarani-Najaran, Z. (2020). Flaxseed for health and disease: Review of clinical trials. Combinatorial Chemistry & High Throughput Screening, 23(8), 699-722.
- van den Brandt, P. A., & Schouten, L. J. (2015). Relationship of tree nut, peanut and peanut butter intake with total and cause-specific mortality: a cohort study and meta-analysis. International journal of epidemiology, 44(3), 1038-1049.

**Mixture of oils – looking into active ingredient**

- Amiri, M., Ghaneian, M. T., Zare-Sakhvidi, M. J., Rahmanian, M., Nadjarzadeh, A., Moghtaderi, F., Raeisi-Dehkordi, H., Zimorovat, A., Jafari, F., Zavar-Reza, J., Jahan-Mihan, A., Reza Aghaei-Meybodi, M., & Salehi-Abargouei, A. (2019). The effect of canola oil compared with sesame and sesame-canola oil on cardio-metabolic biomarkers in patients with type 2 diabetes: Design and research protocol of a randomized, triple-blind, three-way, crossover clinical trial. ARYA atherosclerosis, 15(4), 168–178.
- Rett BS, Whelan J. Increasing dietary linoleic acid does not increase tissue arachidonic acid content in adults consuming Western-type diets: a systematic review. Nutr Metab 2011; 8: 36.
- Wendland E, Farmer A, Glasziou P, et al. Effect of alpha linolenic acid on cardiovascular risk markers: a systematic review. Heart (British Cardiac Society) 2006;92:166–9.
- Derakhshande-Rishehri, S. M., Mansourian, M., Kelishadi, R., & Heidari-Beni, M. (2015). Association of foods enriched in conjugated linoleic acid (CLA) and CLA supplements with lipid profile in human studies: a systematic review and meta-analysis. Public health nutrition, 18(11), 2041–2054
- Jeevan, S., Sindhu, R., Manipal, S., Prabu, D., Mohan, R., & Bharathwaj, V. V. (2019). Efficacy of oil pulling with sesame oil in comparison with other oils and chlorhexidine for oral health: a systematic review. *Journal of Pharmaceutical Sciences and Research*, *11*(11), 3573-3578.
- Ferguson, J. J., Stojanovski, E., MacDonald-Wicks, L., & Garg, M. L. (2016). Fat type in phytosterol products influence their cholesterol-lowering potential: A systematic review and meta-analysis of RCTs. Progress in lipid research, 64, 16–29.
- Schwingshackl L, Strasser B, Hoffmann G. Effects of monounsaturated fatty acids on cardiovascular risk factors: a systematic review and meta-analysis. Ann Nutr Metab. 2011;59:176–186.
- Schwingshackl L, Strasser B, Hoffmann G. Effects of monounsaturated fatty acids on glycaemic control in patients with abnormal glucose metabolism: a systematic review and meta-analysis. Ann Nutr Metab. 2011;58:290–296.
- Harika RK, Eilander A, Alssema M, et al. Intake of fatty acids in general populations worldwide does not meet dietary recommendations to prevent coronary heart disease: a systematic review of data from 40 countries. Ann Nutr Metab. 2013;63:229–238
- Lane, K., Derbyshire, E., Li, W., & Brennan, C. (2014). Bioavailability and potential uses of vegetarian sources of omega-3 fatty acids: a review of the literature. Critical reviews in food science and nutrition, 54(5), 572–579.

**Diet only**

- Esposito K, Maiorino MI, Ceriello A, et al. (2010) Prevention and control of type 2 diabetes by Mediterranean diet: a systematic review. Diabetes Res Clin Pract 89, 97–102.
- Buckland G, Bach A & Serra-Majem L (2008) Obesity and the Mediterranean diet: a systematic review of observational and intervention studies. Obes Rev 9, 582–593.
- Sabate, J., & Fraser, G. E. (1994). Nuts: a new protective food against coronary heart disease. Current Opinion in lipidology, *5*(1), 11-16.
- Sabaté, J., & Wien, M. (2013). Consumption of nuts in the prevention of cardiovascular disease. *Current Nutrition Reports*, *2*(4), 258-266.
- Sofi F, Abbate R, Gensini GF, et al. (2010) Accruing evidence about benefits of adherence to the Mediterranean diet on health: an updated systematic review and meta-analysis. Am J Clin Nutr 92, 1189–1196.
- Tabeshpour, J., Razavi, B. M., & Hosseinzadeh, H. (2017). Effects of avocado (Persea americana) on metabolic syndrome: A comprehensive systematic review. Phytotherapy research, 31(6), 819-837.
- Ulmann, L., Blanckaert, V., Mimouni, V., X Andersson, M., Schoefs, B., & Chénais, B. (2017). Microalgal fatty acids and their implication in health and disease. Mini reviews in medicinal chemistry, 17(12), 1112-1123.
- Parilli-Moser I, Hurtado-Barroso S, Guasch-Ferré M, Lamuela-Raventós RM (2022). Effect of Peanut Consumption on Cardiovascular Risk Factors: A Randomized Clinical Trial and Meta-Analysis. Front Nutr. ;9:853378
- Markellos C, Ourailidou ME, Gavriatopoulou M, Halvatsiotis P, Sergentanis TN, Psaltopoulou T (2022). Olive oil intake and cancer risk: A systematic review and meta-analysis. PLoS One.;17(1):e0261649.

**Other oil which is not cooking oil**

- Jolfaie N, Rouhani M, Surkan P, Siassi F, Azadbakht L. Rice bran oil decreases total and LDL cholesterol in humans: a systematic review and meta-analysis of randomized controlled clinical trials. Horm Metab Res. 2016;48(07):417–426.
- Wendland E, Farmer A, Glasziou P, Neil A. Effect of a linolenic acid on cardiovascular risk markers: a systematic review. Heart 2006;92:166–9.
- Farvid, M. S., M. Ding, A. Pan, Q. Sun, S. E. Chiuve, L. M. Steffen, W. C. Willett, and F. B. Hu. 2014. Dietary linoleic acid and risk of coronary heart disease: a systematic review and meta-analysis of prospective cohort studies. Circulation. 130: 1568–1578.
- Mahmassani, H. A., Avendano, E. E., Raman, G., & Johnson, E. J. (2018). Avocado consumption and risk factors for heart disease: a systematic review and meta-analysis. The American journal of clinical nutrition, 107(4), 523–536
- Fuhong, D., Xiang, G., Haiying, L., Jiangye, W., Xueming, G., & Wenxiao, C. (2018). Evaluation of efficacy and safety for Brucea javanica oil emulsion in the control of the malignant pleural effusions via thoracic perfusion. BMC cancer, 18(1), 1-13.
- Emamat H, Najafpour Boushehri S, Eslami MA, Saneei Totmaj A, Ghalandari H. (2023) The effects of flaxseed or its oil supplementations on polycystic ovary syndrome: A systematic review of clinical trials. Phytother Res.;37(3):1082-1091
- Martínez-González MA, Sayón-Orea C, Bullón-Vela V, Bes-Rastrollo M, Rodríguez-Artalejo F, Yusta-Boyo MJ, García-Solano M (2022). Effect of olive oil consumption on cardiovascular disease, cancer, type 2 diabetes, and all-cause mortality: A systematic review and meta-analysis. Clin Nutr.; 41(12):2659-2682

**Study abstract**

- Shah, S. A., & Yan, F. (2013). Abstract P104: Impact of Avocado Enriched Diets on Serum Lipids: A Meta-Analysis.
- Shortt, C., Musa-Veloso, K., & Poon, T. (2012). Enhanced low density lipoprotein-cholesterol lowering efforts by consuming plant stanols as part of a low saturated fat diet. Proceedings of the Nutrition Society, 71(OCE3).
- Sun, Y., Neelakantan, N., Wu, Y., & Van Dam, R. M. (2014). Effects of palm oil consumption on blood lipids: A meta-analysis of clinical trials. In Circulation Conference: American Heart Association’s Epidemiology and Prevention/Nutrition, Physical Activity, and Metabolism (Vol. 129).

**Appendix Figure 1: AMSTAR-2 (measurement tool for assessing systematic reviews, version 2) ratings, by item**

**Appendix Figure 2: Individual and overall rating of each included review**

|  | **AMSTAR-2** | | | | | | | | | | | | | | | |  |
| --- | --- | --- | --- | --- | --- | --- | --- | --- | --- | --- | --- | --- | --- | --- | --- | --- | --- |
| **Author Year** | **Q1** | **Q2** | **Q3** | **Q4** | **Q5** | **Q6** | **Q7** | **Q8** | **Q9** | **Q10** | **Q11** | **Q12** | **Q13** | **Q14** | **Q15** | **Q16** | **Overall rating** |
| Al-Ghamady 2018 |  |  |  |  |  |  |  |  |  |  |  |  |  |  |  |  | Low |
| Amiri 2020 |  |  |  |  |  |  |  |  |  |  |  |  |  |  |  |  | High |
| Atefi 2022 |  |  |  |  |  |  |  |  |  |  |  |  |  |  |  |  | Low |
| Azad 2021 |  |  |  |  |  |  |  |  |  |  |  |  |  |  |  |  | Moderate |
| Basch 2007 |  |  |  |  |  |  |  |  |  |  |  |  |  |  |  |  | Low |
| Cardoso 2016 |  |  |  |  |  |  |  |  |  |  |  |  |  |  |  |  | Low |
| Cortez-Ribeiro 2023 |  |  |  |  |  |  |  |  |  |  |  |  |  |  |  |  | Critically low |
| Dhanasekara 2022 |  |  |  |  |  |  |  |  |  |  |  |  |  |  |  |  | Critically low |
| Dong 2017 |  |  |  |  |  |  |  |  |  |  |  |  |  |  |  |  | Low |
| Duarte 2022 |  |  |  |  |  |  |  |  |  |  |  |  |  |  |  |  | Low |
| Eyres 2016 |  |  |  |  |  |  |  |  |  |  |  |  |  |  |  |  | Low |
| Fattore 2014 |  |  |  |  |  |  |  |  |  |  |  |  |  |  |  |  | Low |
| George 2019 |  |  |  |  |  |  |  |  |  |  |  |  |  |  |  |  | Low |
| Ghobadi 2019a (Canola) |  |  |  |  |  |  |  |  |  |  |  |  |  |  |  |  | Low |
| Ghobadi 2019b (Olive) |  |  |  |  |  |  |  |  |  |  |  |  |  |  |  |  | Low |
| Harland 2009 |  |  |  |  |  |  |  |  |  |  |  |  |  |  |  |  | Critically low |
| Hisham 2020 |  |  |  |  |  |  |  |  |  |  |  |  |  |  |  |  | Low |
| Hohmann 2015 |  |  |  |  |  |  |  |  |  |  |  |  |  |  |  |  | Moderate |
| Ismail 2018 |  |  |  |  |  |  |  |  |  |  |  |  |  |  |  |  | Moderate |
| Jayawarderna 2020 |  |  |  |  |  |  |  |  |  |  |  |  |  |  |  |  | low |
| Jolfaie 2016 |  |  |  |  |  |  |  |  |  |  |  |  |  |  |  |  | Low |
| Khalesi 2015 |  |  |  |  |  |  |  |  |  |  |  |  |  |  |  |  | Critically low |
| Lin 2013 |  |  |  |  |  |  |  |  |  |  |  |  |  |  |  |  | Low |
| Ma & Lee 2016 |  |  |  |  |  |  |  |  |  |  |  |  |  |  |  |  | Low |
| Mahmudiono 2022 |  |  |  |  |  |  |  |  |  |  |  |  |  |  |  |  | Low |
| Martínez-Gonzále 2014 |  |  |  |  |  |  |  |  |  |  |  |  |  |  |  |  | Low |
| Mohammadi-Sartang 2017 |  |  |  |  |  |  |  |  |  |  |  |  |  |  |  |  | Low |
| Neelatakan 2020 |  |  |  |  |  |  |  |  |  |  |  |  |  |  |  |  | Moderate |
| Pan 2009 |  |  |  |  |  |  |  |  |  |  |  |  |  |  |  |  | Critically low |
| Pourrajab 2021 |  |  |  |  |  |  |  |  |  |  |  |  |  |  |  |  | Moderate |
| Pourrajab 2022 |  |  |  |  |  |  |  |  |  |  |  |  |  |  |  |  | Moderate |
| Psaltopoulou 2011 |  |  |  |  |  |  |  |  |  |  |  |  |  |  |  |  | Critically low |
| Raeisi-Dehkordi 2019 |  |  |  |  |  |  |  |  |  |  |  |  |  |  |  |  | Low |
| Ren 2016 |  |  |  |  |  |  |  |  |  |  |  |  |  |  |  |  | Critically low |
| Schwingshackl 2015 |  |  |  |  |  |  |  |  |  |  |  |  |  |  |  |  | Low |
| Schwingshackl 2017 |  |  |  |  |  |  |  |  |  |  |  |  |  |  |  |  | Low |
| Schwingshackl 2014 |  |  |  |  |  |  |  |  |  |  |  |  |  |  |  |  | Low |
| Sekhar 2022 |  |  |  |  |  |  |  |  |  |  |  |  |  |  |  |  | Critically low |
| Sun 2015 |  |  |  |  |  |  |  |  |  |  |  |  |  |  |  |  | Low |
| Swarnamali 2023 |  |  |  |  |  |  |  |  |  |  |  |  |  |  |  |  | Critically low |
| Teng 2020 |  |  |  |  |  |  |  |  |  |  |  |  |  |  |  |  | Moderate |
| Ursoniu 2016 |  |  |  |  |  |  |  |  |  |  |  |  |  |  |  |  | Low |
| Ursoniu 2019 |  |  |  |  |  |  |  |  |  |  |  |  |  |  |  |  | Low |
| Vittori Gouveia 2016 |  |  |  |  |  |  |  |  |  |  |  |  |  |  |  |  | Low |
| Voon 2019 |  |  |  |  |  |  |  |  |  |  |  |  |  |  |  |  | Low |
| Wang 2019 |  |  |  |  |  |  |  |  |  |  |  |  |  |  |  |  | Low |
| Zamora 2018 |  |  |  |  |  |  |  |  |  |  |  |  |  |  |  |  | Low |
| Zulkiply 2019 |  |  |  |  |  |  |  |  |  |  |  |  |  |  |  |  | Critically low |

**Legend**

|  | **Yes** |
| --- | --- |
|  | **Partial yes** |
|  | **No** |
|  | **No meta-analysis** |

**Supplementary Table S1: Estimated effect sizes of canola oil consumption on health outcomes**

| Source | Outcome | Oil type | Comparator | No of studies | Sample size | Random effect size (95% CI) | *I*^2^,% | GRADE rating |
| --- | --- | --- | --- | --- | --- | --- | --- | --- |
| Harland 2009 | Total cholesterol (mmol/L) | Canola oil | Olive oil, sunflower oil, palm oil, corn oil, safflower oil, soybean oil | 10 | 222 | –0.86 (-1.05 to -0.66) | 0 | ⨁◯◯◯  Very low |
| Ghobadi 2019a | Total cholesterol (mmol/L) | Canola oil | Sunflower oil, rice bran oil, olive oil, safflower oil, corn oil, soybean oil, palm oil, coconut oil | 26 | 589 | -0.15 (-0.30 to -0.02) | 57 | ⨁⨁◯◯  Low |
| Amiri 2020 | Total cholesterol (mmol/L) | Canola oil | Olive oil, palm oil, sunflower oil, soybean oil, safflower oil, coconut oil, rice bran oil, flaxseed oil | 37 | 1,637 | -0.27 (-0.37 to -0.17) | 92.4 | ⨁⨁⨁◯  Moderate |
| Pourrajab 2022 | Total cholesterol (mmol/L) | Canola oil | Olive oil | 12 | 504 | -0.23 (-0.35 to -0.11) | 0 | ⨁⨁⨁⨁  High |
| Ghobadi 2019a | Triglyceride  (mmol/L) | Canola oil | Sunflower oil, rice bran oil, olive oil, safflower oil, corn oil, soybean oil, palm oil, coconut oil | 24 | 463 | -0.02 (-0.10 to 0.06) | 40.7 | ⨁◯◯◯  Very low |
| Amiri 2020 | Triglyceride  (mmol/L) | Canola oil | Olive oil, palm oil, sunflower oil, soybean oil, safflower oil, coconut oil, rice bran oil, flaxseed oil | 37 | 1,647 | -0.02 (-0.05 to 0.02) | 76.2 | ⨁⨁◯◯  Low |
| Pourrajab 2022 | Triglyceride  (mmol/L) | Canola oil | Olive oil | 12 | 522 | -0.07 (-0.14 to 0.01) | 0 | ⨁⨁⨁◯  Moderate |
| Harland 2009 | LDL (mmol/L) | Canola oil | Olive oil, sunflower oil, palm oil, corn oil, safflower oil, soybean oil | 9 | 204 | –0.87 (-1.07 to -0.66) | 0 | ⨁◯◯◯  Very low |
| Ghobadi 2019a | LDL (mmol/L) | Canola oil | Sunflower oil, rice bran oil, olive oil, safflower oil, corn oil, soybean oil, palm oil, coconut oil | 25 | 573 | -0.17 (-0.28 to -0.02) | 63.3 | ⨁⨁◯◯  Low |
| Amiri 2020 | LDL (mmol/L) | Canola oil | Olive oil, palm oil, sunflower oil, soybean oil, safflower oil, coconut oil, rice bran oil, flaxseed oil | 35 | 1,569 | -0.23 (-0.33 to -0.14) | 91.5 | ⨁⨁⨁◯  Moderate |
| Pourrajab 2022 | LDL (mmol/L) | Canola oil | Olive oil | 12 | 504 | -0.16 (-0.25 to -0.06) | 0 | ⨁⨁⨁⨁  High |
| Ghobadi 2019a | HDL (mmol/L) | Canola oil | Sunflower oil, rice bran oil, olive oil, safflower oil, corn oil, soybean oil, palm oil, coconut oil | 26 | 589 | 0.01 (-0.03 to 0.02) | 0 | ⨁⨁◯◯  Low |
| Amiri 2020 | HDL (mmol/L) | Canola oil | Olive oil, palm oil, sunflower oil, soybean oil, safflower oil, coconut oil, rice bran oil, flaxseed oil | 36 | 1,644 | -0.01 (-0.02 to 0.00) | 39.1 | ⨁⨁⨁◯  Moderate |
| Pourrajab 2022 | HDL (mmol/L) | Canola oil | Olive oil | 12 | 522 | -0.02 (-0.06 to 0.01) | 0 | ⨁⨁⨁◯  Moderate |
| Amiri 2020 | VLDL (mmol/L) | Canola oil | Olive oil, palm oil, sunflower oil, soybean oil | 14 | 404 | -0.001 (-0.05 to 0.04) | 64.8 | ⨁◯◯◯  Very low |
| Pourrajab 2022 | VLDL (mmol/L) | Canola oil | Olive oil | 4 | 160 | -0.02 (−0.13 to 0.10) | 71.1 | ⨁◯◯◯  Very low |
| Ghobadi 2019a | LDL/HDL | Canola oil | Sunflower oil, olive oil, safflower oil, palm oil | 7 | 455 | -0.17 (-0.38 to 0.05) | 17.2 | ⨁◯◯◯  Very low |
| Amiri 2020 | LDL/HDL | Canola oil | Olive oil, palm oil, sunflower oil, safflower oil | 10 | 526 | -0.21 (-0.34 to -0.08) | 23.7 | ⨁⨁⨁◯  Moderate |
| Pourrajab 2022 | LDL/HDL | Canola oil | Olive oil | 4 | 180 | -0.30 ( -0.53 to −0.06) | 0 | ⨁⨁◯◯  Low |
| Ghobadi 2019a | TC/HDL | Canola oil | Sunflower oil, olive oil, safflower oil, corn oil, soybean oil, palm oil, coconut oil | 8 | 330 | -0.07 (-0.3 to 0.15) | 23.2 | ⨁◯◯◯  Very low |
| Amiri 2020 | TC/HDL | Canola oil | Palm oil, soybean oil, safflower oil, coconut oil | 15 | 753 | -0.13 (-0.21 to -0.06) | 12.3 | ⨁⨁⨁◯  Moderate |
| Pourrajab 2022 | TC/HDL | Canola oil | Olive oil | 6 | 224 | -0.13 (-0.31 to 0.05) | 0 | ⨁⨁⨁◯  Moderate |
| Amiri 2020 | Triglycerides in HDL | Canola oil | Olive oil, sunflower oil | 7 | 217 | 0.00 (-0.01 to 0.01) | 41.8 | ⨁⨁◯◯  Low |
| Amiri 2020 | Triglycerides in LDL | Canola oil | Olive oil, sunflower oil | 8 | 325 | -0.01 (-0.04 to 0.01) | 74.3 | ⨁⨁◯◯  Low |
| Amiri 2020 | Triglycerides in VLDL | Canola oil | Olive oil, sunflower oil | 8 | 325 | 0.06 (-0.03 to 0.14) | 48.0 | ⨁⨁◯◯  Low |
| Ghobadi 2019a | Apolipoprotein A-1 (g/L) | Canola oil | Sunflower oil, olive oil, safflower oil, corn oil, soybean oil, palm oil | 12 | 498 | 0.05 (-0.02 to 0.12 ) | 78.8 | ⨁◯◯◯  Very low |
| Amiri 2020 | Apolipoprotein A-1 (g/L) | Canola oil | Olive oil, palm oil, sunflower oil, soybean oil, safflower oil, flaxseed oil | 17 | 811 | 0.01 (-0.02 to 0.04) | 60.7 | ⨁⨁◯◯  Low |
| Ghobadi 2019a | Apolipoprotein B (g/L) | Canola oil | Sunflower oil, olive oil, safflower oil, corn oil, soybean oil, palm oil | 10 | 451 | -0.03 (-0.07 to 0.01 ) | 33.5 | ⨁◯◯◯  Very low |
| Amiri 2020 | Apolipoprotein B (g/L) | Canola oil | Olive oil, palm oil, sunflower oil, soybean oil, safflower oil, flaxseed oil | 14 | 746 | -0.03 (-0.06 to -0.01) | 52.0 | ⨁⨁⨁◯  Moderate |
| Amiri 2020 | Apolipoprotein B to Apolipoprotein A-1 ratio | Canola oil | Safflower oil, coconut oil, flaxseed oil | 6 | 405 | -0.02 (-0.03 to -0.01) | 0 | ⨁⨁◯◯  Low |
| Amiri 2020 | Lipoprotein (g/L) | Canola oil | Olive oil, palm oil, sunflower oil, safflower oil | 8 | 262 | 0.03 (-0.04 to 0.10) | 0 | ⨁⨁⨁◯  Moderate |
| Amiri 2020 | Fasting blood sugar (mmol/L) | Canola oil | Olive oil, palm oil, sunflower oil, soybean oil | 13 | 612 | -0.07 (-0.15 to 0.02) | 0 | ⨁⨁⨁◯  Moderate |
| Amiri 2020 | Insulin (μU/mL) | Canola oil | Olive oil, palm oil, sunflower oil, soybean oil | 12 | 469 | -0.02 (-0.59 to 0.54) | 21.9 | ⨁⨁⨁◯  Moderate |
| Amiri 2020 | HOMA-IR | Canola oil | Olive oil, palm oil, sunflower oil, soybean oil | 4 | 147 | -0.21 (-0.56 to 0.14) | 0 | ⨁⨁◯◯  Low |
| Amiri 2020 | Systolic blood pressure (mmHg) | Canola oil | Olive oil, palm oil, sunflower oil, soybean oil, flaxseed oil | 8 | 490 | -0.70 (-2.36 to 0.97) | 13.9 | ⨁⨁⨁◯  Moderate |
| Amiri 2020 | Diastolic blood pressure (mmHg) | Canola oil | Olive oil, palm oil, sunflower oil, soybean oil, flaxseed oil | 7 | 486 | -0.27 (-1.39 to 0.85) | 0 | ⨁⨁⨁◯  Moderate |
| Amiri 2020 | c-reactive protein (mg/L) | Canola oil | Olive oil, sunflower oil | 3 | 96 | 0.34 (-0.39 to 1.06) | 0 | ⨁◯◯◯  Very low |
| Raeisi-Dehkordi 2019 | Body weight (kg) | Canola oil | Rapeseed oil, sunflower oil, olive oil, safflower oil | 23 | 1078 | -0.30 (-0.52 to -0.08) | 0 | ⨁⨁⨁◯  Moderate |
| Raeisi-Dehkordi 2019 | Body mass index (kg/m^2^) | Canola oil | Sunflower oil, rapeseed oil, flaxseed oil | 12 | 577 | -0.07 (-0.27 to 0.12) | 0 | ⨁⨁⨁◯  Moderate |
| Raeisi-Dehkordi 2019 | Waist circumference (cm) | Canola oil | Rapeseed oil, sunflower oil, flaxseed oil | 6 | 481 | 3.15 (-1.67 to 7.98) | 88.3 | ⨁⨁◯◯  Low |
| Raeisi-Dehkordi 2019 | Body fat (%) | Canola oil | Rapeseed oil, sunflower oil, flaxseed oil | 4 | 247 | -0.04 (-0.19 to 0.10) | 0 | ⨁⨁◯◯  Low |
| Raeisi-Dehkordi 2019 | Waist hip ratio | Canola oil | Rapeseed oil, sunflower oil, flaxseed oil | 6 | 261 | 0 (-0.01 to 0.01) | 0 | ⨁⨁⨁◯  Moderate |
| Raeisi-Dehkordi 2019 | Android to gynoid fat ratio | Canola oil | Rapeseed oil, sunflower oil, flaxseed oil | 2 | 148 | -0.01 (-0.02 to 0.01) | 0 | ⨁◯◯◯  Very low |
| Raeisi-Dehkordi 2019 | Hip circumference (cm) | Canola oil | Rapeseed oil, sunflower oil, flaxseed oil | 3 | 196 | -0.24 (-3.01 to 2.54) | 0 | ⨁◯◯◯  Very low |
| Raeisi-Dehkordi 2019 | Lean mass (kg) | Canola oil | Rapeseed oil, sunflower oil, flaxseed oil | 2 | 182 | 0.01 (-0.17 to 0.19) | 0 | ⨁◯◯◯  Very low |

**Supplementary Table S 2: Estimated effect sizes of peanut oil consumption on health outcomes**

| Source | Outcome | Oil type | Comparator | No of studies | Sample size | Random effect size (95% CI) | *I*^2^,% | GRADE rating |
| --- | --- | --- | --- | --- | --- | --- | --- | --- |
| Azad 2021 | Total cholesterol  (mmol/L) | Peanut oil | Canola oil, sesame oil, safflower oil, sunflower oil, olive oil | 3 | 203 | 0.08 (-0.10 to 0.25) | 0 | ⨁◯◯◯  Very low |
| Azad 2021 | Triglyceride (mmol/L) | Peanut oil | Canola oil, sesame oil, safflower oil, sunflower oil, olive oil | 3 | 203 | 0.02 (-0.17 to 0.22) | 0 | ⨁◯◯◯  Very low |
| Azad 2021 | LDL (mmol/L) | Peanut oil | Canola oil, sesame oil, safflower oil, sunflower oil, olive oil | 3 | 203 | -0.02 (-0.18 to 0.14) | 0 | ⨁◯◯◯  Very low |
| Azad 2021 | HDL (mmol/L) | Peanut oil | Canola oil, sesame oil, safflower oil, sunflower oil, olive oil | 3 | 203 | 0.06 (0.01 to 0.11) | 33.6 | ⨁◯◯◯  Very low |
| Azad 2021 | Weight (kg) | Peanut oil | Milk, safflower oil, olive oil | 2 | 128 | 0.90 (0.40 to 1.40) | 0 | ⨁◯◯◯  Very low |
| Azad 2021 | Systolic blood pressure (mmHg) | Peanut oil | Sesame oil, safflower oil, sunflower oil, olive oil | 3 | 167 | 0.15 (-6.37 to 6.67) | 71.1 | ⨁◯◯◯  Very low |
| Azad 2021 | Diastolic blood pressure (mmHg) | Peanut oil | Sesame oil, safflower oil, sunflower oil, olive oil | 3 | 167 | 0.24 (-3.05 to 3.53) | 48.8 | ⨁◯◯◯  Very low |

**Supplementary Table S 3: Estimated effect sizes of flaxseed oil consumption on health outcomes**

| Source | Outcome | Oil type | Comparator | No of studies | Sample size | Random effect size (95% CI) | *I*^2^,% | GRADE rating |
| --- | --- | --- | --- | --- | --- | --- | --- | --- |
| Pan 2009 | Total cholesterol (mmol/L) | Flaxseed oil | Hempseed oil, coconut oil, sunflower oil, safflower oil, olive oil, fish oil | 13 | 525 | -0.01 (-0.04 to 0.03) | 0 | ⨁◯◯◯  Very low |
| Pan 2009 | LDL (mmol/L) | Flaxseed oil | Hempseed oil, coconut oil, sunflower oil, safflower oil, olive oil, fish oil | 12 | 490 | 0.07 (-0.04 to 0.17) | 0 | ⨁◯◯◯  Very low |
| Pan 2009 | HDL (mmol/L) | Flaxseed oil | Hempseed oil, coconut oil, sunflower oil, safflower oil, olive oil, fish oil | 13 | 525 | -0.01 (-0.04 to 0.03) | 0 | ⨁◯◯◯  Very low |
| Pan 2009 | Triglyceride (mmol/L) | Flaxseed oil | Hempseed oil, coconut oil, sunflower oil, safflower oil, olive oil, fish oil | 12 | 497 | -0.05 (-0.13 to 0.33) | 0 | ⨁◯◯◯  Very low |
| Ren 2016 | c-reactive protein (mg/L) | Flaxseed oil | Soybean oil; olive oil; safflower oil | 8 | 398 | -0.39 (-0.10 to 0.87) | 55.6 | ⨁⨁◯◯  Low |
| Ursoniu 2019 | c-reactive protein (mg/L) | Flaxseed oil | Olive oil, sunflower oil | 4 | 277 | -0.86 (-2.93 to 1.21) | 88.1 | ⨁◯◯◯  Very low |
| Mohammadi-Sartang 2017 | Body weight (kg) | Flaxseed oil | Sunflower oil, soybean oil, corn oil, olive oil, safflower oil | 8 | 309 | -0.37 (-1.49 to 0.74) | 0 | ⨁⨁◯◯  Low |
| Mohammadi-Sartang 2017 | Body mass index (kg/m^2^) | Flaxseed oil | Sunflower oil, corn oil, safflower oil, olive oil, canola oil | 17 | 784 | 0.00 (-0.09 to 0.09) | 0 | ⨁⨁◯◯  Low |
| Mohammadi-Sartang 2017 | Waist circumference (cm) | Flaxseed oil | Soybean oil, olive oil | 6 | 348 | -0.05 (-1.07 to 0.96) | 0 | ⨁◯◯◯  Very low |
| Ursoniu 2016 | Systolic blood pressure (mmHg) | Flaxseed oil | Sunflower oil; safflower oil; olive oil | 4 | 178 | -4.62 (-11.86 to 2.62) | 71.0 | ⨁◯◯◯  Very low |
| Khalesi 2015 | Systolic blood pressure (mmHg) | Flaxseed oil | Sunflower oil, soybean oil, hempseed oil | 3 | 86 | -1.44 (-4.47 to 1.60) | 0 | ⨁◯◯◯  Very low |
| Mahmudiono 2022 | Systolic blood pressure  (mmHg) | Flaxseed oil | Sunflower oil; Soybean oil; Safflower oil; Corn oil | 5 | 235 | -3.86 (-7.59 to -0.13) | 51.3 | ⨁⨁◯◯  Low |
| Ursoniu 2016 | Diastolic blood pressure (mmHg) | Flaxseed oil | Sunflower oil; safflower oil; olive oil | 4 | 178 | -4.10 (-6.81 to -1.39) | 16.0 | ⨁◯◯◯  Very low |
| Khalesi 2015 | Diastolic blood pressure  (mmHg) | Flaxseed oil | Sunflower oil, soybean oil, hempseed oil | 3 | 86 | -0.38 (-2.79 to 2.03) | 0 | ⨁◯◯◯  Very low |
| Mahmudiono 2022 | Diastolic blood pressure  (mmHg) | Flaxseed oil | Sunflower oil; Soybean oil; Safflower oil; Corn oil | 5 | 235 | -1.71 (-3.67 to 0.26) | 36.4 | ⨁⨁◯◯  Low |

**Supplementary Table S 4: Estimated effect sizes of olive oil consumption on health outcomes**

| Source | Outcome | Oil type | Comparator | No of studies | Sample size | Random effect size (95% CI) | *I*^2^,% | GRADE rating |
| --- | --- | --- | --- | --- | --- | --- | --- | --- |
| Ghobadi 2019b | Total cholesterol (mmol/L) | Olive oil | Soybean oil, palm oil, corn oil, rapeseed oil, safflower oil, sesame oil, flaxseed oil, rice bran oil | 30 | 1,144 | 0.19 (0.11 to 0.28) | 38.3 | ⨁⨁◯◯  Low |
| Ghobadi 2019b | Triglyceride  (mmol/L) | Olive oil | Soybean oil, palm oil, corn oil, rapeseed oil, safflower oil, sesame oil, flaxseed oil, rice bran oil | 24 | 956 | 0.11 (0.01 to 0.21) | 0 | ⨁⨁◯◯  Low |
| Ghobadi 2019b | LDL  (mmol/L) | Olive oil | Soybean oil, palm oil, corn oil, rapeseed oil, safflower oil, sesame oil, flaxseed oil, rice bran oil | 23 | 969 | 0.11 (0.04 to 0.18) | 22.9 | ⨁⨁◯◯  Low |
| Ghobadi 2019b | HDL (mmol/L) | Olive oil | Soybean oil, palm oil, corn oil, rapeseed oil, safflower oil, sesame oil, flaxseed oil, rice bran oil | 25 | 1,014 | 0.04 (0.01 to 0.06) | 0.0 | ⨁⨁◯◯  Low |
| Ghobadi 2019b | Apolipoprotein A1 (g/L) | Olive oil | Flaxseed oil, soybean oil, corn oil, rapeseed oil, peanut oil, sunflower oil | 10 | 308 | 0.04 (-0.004 to 0.09) | 36.4 | ⨁◯◯◯  Very low |
| Ghobadi 2019b | Apolipoprotein B (g/L) | Olive oil | Flaxseed oil, soybean oil, corn oil, rapeseed oil, peanut oil, sunflower oil | 10 | 308 | 0.03 (-0.01 to 0.09) | 38.6 | ⨁◯◯◯  Very low |
| Schwingshack 2017 | HbA1c (%) | Olive oil | Sunflower oil, corn oil, palm oil | 10 | 495 | -0.35 (-0.47 to -0.22) | 0.0 | ⨁⨁◯◯  Low |
| Schwingshack 2017 | Fasting blood sugar (mmol/L) | Olive oil | Sunflower oil, corn oil, palm oil | 12 | 514 | -0.54 (-0.87 to -0.21) | 37.5 | ⨁⨁◯◯  Low |
| Schwingshack 2015 | c-reactive protein (mg/L) | Olive oil | Flaxseed oil, sunflower oil, coconut oil | 3 | 122 | -0.22 (-0.52 to 0.09) | 0 | ⨁◯◯◯  Very low |
| Zamora 2018 | Body weight (kg) | Olive oil | Canola oil, corn oil | 2 | 104 | -2.19 (-7.06 to 2.67) | 80.6 | ⨁◯◯◯  Very low |
| Zamora 2018 | Body mass index (kg/m^2^) | Olive oil | Canola oil, corn oil, palm oil | 3 | 264 | -0.42 (-0.92 to 0.08) | 91.7 | ⨁◯◯◯  Very low |
| Zamora 2018 | Waist circumference (cm) | Olive oil | Canola oil, corn oil | 2 | 104 | -1.25 (-4.06 to 1.57) | 64.7 | ⨁◯◯◯  Very low |
| Schwingshackl 2014 | Coronary heart disease | Olive oil | NR | 4 | NR | 0.80 (0.57 to 1.14) | 77.0 | ⨁◯◯◯  Very low |
| Al-Ghamady 2018 | Cardiovascular events | Olive oil | Regular low fat diet | 2 | 14,663 | RR: 0.97 (0.68 to 1.38) | 76.0 | ⨁◯◯◯  Very low |
| Schwingshackl 2014 | Cardiovascular mortality | Olive oil | NR | 5 | NR | RR: 0.70 (0.48 to 1.03) | 71.0 | ⨁◯◯◯  Very low |
| Al-Ghamady 2018 | Cardiovascular mortality | Olive oil | Regular low fat diet | 2 | 14,663 | RR: 1.07 (0.77 to 1.48) | 0 | ⨁◯◯◯  Very low |
| Schwingshackl 2014 | Combined cardiovascular events | Olive oil | NR | 7 | NR | 0.72 (0.57 to 0.91) | 75.0 | ⨁◯◯◯  Very low |
| Schwingshackl 2014 | All-cause mortality | Olive oil | NR | 5 | NR | RR: 0.77 (0.71 to 0.84) | 0 | ⨁◯◯◯  Very low |
| Al-Ghamady 2018 | All-cause mortality | Olive oil | Regular low fat diet | 4 | 22,114 | RR: 0.99 (0.85 to 1.15) | 0 | ⨁◯◯◯  Very low |
| Schwingshack 2015 | Interleukin-6 (pg/mL) | Olive oil | Palm oil, coconut oil | 1 | 45 | -0.50 (-2.94 to 1.93) | NA | ⨁◯◯◯  Very low |
| Martinez Gonzalez 2014 | Risk of developing chronic heart disease | Diet supplemented with olive oil | Reduced fat diet, usual diet | 7 | 3169 | RR: 0.80 (0.60 to 0.90) | 85.4 | ⨁◯◯◯  Very low |
| Schwingshackl 2014 | Risk of developing stroke | Olive oil | NR | 2 | NR | RR: 0.60 (0.47 to 0.77) | 0 | ⨁◯◯◯  Very low |
| Martinez Gonzalez 2014 | Risk of developing stroke | Diet supplemented with olive oil | Reduced fat diet, usual diet | 3 | 650 | RR: 0.76 (0.66 to 0.85) | 0 | ⨁◯◯◯  Very low |
| Psaltopoulou 2011 | Risk of developing breast cancer | Diet supplemented with olive oil | NR | 5 | NR | OR: 0.64 (0.47 to 0.89) | NR | ⨁◯◯◯  Very low |
| Psaltopoulou 2011 | Risk of developing digestive cancer | Diet supplemented with olive oil | NR | 8 | NR | OR: 0.70 (0.61 to 0.81) | NR | ⨁◯◯◯  Very low |
| Psaltopoulou 2011 | Risk of developing other cancer | Diet supplemented with olive oil | NR | 6 | NR | OR: 0.66 (0.55 to 0.79) | NR | ⨁◯◯◯  Very low |

**Supplementary Table S 5: Estimated effect sizes of extra virgin olive oil consumption on health outcomes**

| Source | Outcome | Oil type | Comparator | No of studies | Sample size | Random effect size (95% CI) | *I*^2^,% | GRADE rating |
| --- | --- | --- | --- | --- | --- | --- | --- | --- |
| Hohmann 2015 | Total cholesterol (mmol/L) | Extra virgin olive oil | Olive oil | 6 | 400 | -0.06 (-0.17 to 0.05) | 0 | ⨁◯◯◯  Very low |
| George 2019 | Total cholesterol (mmol/L) | Extra virgin olive oil | Olive oil | 8 | 476 | -0.11 (-0.17 to -0.06) | 0.0 | ⨁◯◯◯  Very low |
| Hohmann 2015 | LDL (mmol/L) | Extra virgin olive oil | Olive oil | 6 | 400 | -0.03 (-0.15 to 0.08) | 38.5 | ⨁◯◯◯  Very low |
| George 2019 | LDL (mmol/L) | Extra virgin olive oil | Olive oil | 8 | 476 | -0.09 (-0.19 to 0.00) | 66.4 | ⨁◯◯◯  Very low |
| Hohmann 2015 | HDL (mmol/L) | Extra virgin olive oil | Olive oil | 6 | 400 | -0.03 (-0.14 to 0.08) | 32.5 | ⨁◯◯◯  Very low |
| George 2019 | HDL (mmol/L) | Extra virgin olive oil | Olive oil | 8 | 522 | 0.07 (0.01 to 0.13) | 64.4 | ⨁◯◯◯  Very low |
| Hohmann 2015 | Triglyceride | Extra virgin olive oil | Olive oil | 5 | 360 | 0.02 (-0.22 to 0.25) | 77.8 | ⨁◯◯◯  Very low |
| Hohmann 2015 | Oxidised LDL (μmol/L) | Extra virgin olive oil | Olive oil | 4 | 300 | -0.25 (-0.50 to 0.00) | 80.0 | ⨁◯◯◯  Very low |
| George 2019 | Oxidised LDL (μmol/L) | Extra virgin olive oil | Olive oil | 5 | 286 | -0.44 (-0.78 to -0.10) | 48.6 | ⨁◯◯◯  Very low |
| Hohmann 2015 | Systolic blood pressure (mmHg) | Extra virgin olive oil | Olive oil | 2 | 70 | -0.52 (-0.77 to -0.27) | 34.0 | ⨁◯◯◯  Very low |
| George 2019 | Systolic blood pressure (mmHg) | Extra virgin olive oil | Olive oil | 3 | NR | -2.03 (-6.57 to 2.50) | 79 | ⨁◯◯◯  Very low |
| Hohmann 2015 | Diastolic blood pressure (mmHg) | Extra virgin olive oil | Olive oil | 2 | 69 | -0.20 (-1.01 to 0.62) | 93.7 | ⨁◯◯◯  Very low |
| George 2019 | Diastolic blood pressure (mmHg) | Extra virgin olive oil | Olive oil | 3 | NR | -2.70 (-5.71 to 0.31) | 78 | ⨁◯◯◯  Very low |
| Hohmann 2015 | Malondialdehyde levels (μmol/L) | Extra virgin olive oil | Olive oil | 2 | 71 | -0.04 (-0.27 to 0.19) | 40.1 | ⨁◯◯◯  Very low |
| George 2019 | Malondialdehyde levels (μmol/L) | Extra virgin olive oil | Olive oil | 3 | 192 | -0.08 (-0.12 to -0.03) | 86.9 | ⨁◯◯◯  Very low |
| George 2019 | Total antioxidant capacity | Extra virgin olive oil | Olive oil | 3 | 73 | 0.30 (-0.26 to 0.86) | 67 | ⨁◯◯◯  Very low |
| George 2019 | Glutathione  peroxidase levels | Extra virgin olive oil | Olive oil | NR | NR | -0.04 (-0.69 to 0.61) | 75 | ⨁◯◯◯  Very low |

**Supplementary Table S 6: Estimated effect sizes of rice bran oil consumption on health outcomes**

| Source | Outcome | Oil type | Comparator | No of studies | Sample size | Random effect size (95% CI) | *I*^2^,% | GRADE rating |
| --- | --- | --- | --- | --- | --- | --- | --- | --- |
| Jolfaie 2016 | Total cholesterol  (mmol/L) | Rice bran oil | Peanut oil, olive oil, corn oil, canola oil, palm oil, sunflower oil | 11 | 313 | -0.33 (-0.47 to -0.19) | 41.0 | ⨁⨁⨁◯  Moderate |
| Pourrajab 2021 | Total cholesterol (mmol/L) | Rice bran oil | Peanut oil, olive oil, virgin olive oil, corn oil, canola oil, palm oil, soybean oil, sunflower oil | 7 | 429 | -0.18 (-0.29 to -0.09) | 30.4 | ⨁⨁◯◯  Low |
| Jolfaie 2016 | Triglyceride (mmol/L) | Rice bran oil | Peanut oil, olive oil, corn oil, canola oil, palm oil, sunflower oil | 11 | 313 | 0.01 (-0.12 to 0.15) | 30.2 | ⨁⨁◯◯  Low |
| Pourrajab 2021 | Triglycerides (mmol/L) | Rice bran oil | Peanut oil, olive oil, virgin olive oil, corn oil, canola oil, palm oil, soybean oil, sunflower oil | 7 | 429 | -0.23 (-0.34 to -0.13) | 16.4 | ⨁⨁◯◯  Low |
| Jolfaie 2016 | LDL (mmol/L) | Rice bran oil | Peanut oil, olive oil, corn oil, canola oil, sunflower oil | 10 | 292 | -0.18 (-0.27 to -0.09) | 59.4 | ⨁⨁⨁◯  Moderate |
| Pourrajab 2021 | LDL (mmol/L) | Rice bran oil | Peanut oil, olive oil, virgin olive oil, corn oil, canola oil, palm oil, soybean oil, sunflower oil | 7 | 429 | 0.01 (-0.04 to 0.05) | 27.3 | ⨁⨁⨁◯  Moderate |
| Jolfaie 2016 | HDL (mmol/L) | Rice bran oil | Peanut oil, olive oil, corn oil, canola oil, sunflower oil | 10 | 292 | 0.02 (-0.02 to 0.05) | 50.4 | ⨁⨁◯◯  Low |
| Pourrajab 2021 | HDL (mmol/L) | Rice bran oil | Peanut oil, olive oil, virgin olive oil, corn oil, canola oil, palm oil, soybean oil, sunflower oil | 7 | 429 | -0.13 (-0.25 to -0.01) | 51.0 | ⨁⨁◯◯  Low |
| Jolfaie 2016 | VLDL (mmol/L) | Rice bran oil | Peanut oil, olive oil, corn oil, canola oil | 3 | 54 | 0.0 (-0.01 to 0.02) | 71.1 | ⨁◯◯◯  Very low |
| Jolfaie 2016 | LDL/HDL | Rice bran oil | Peanut oil, olive oil, corn oil, canola oil | 2 | 80 | 0.08 (-0.22 to 0.07) | 13.0 | ⨁◯◯◯  Very low |
| Jolfaie 2016 | TC/HDL | Rice bran oil | Peanut oil, olive oil, corn oil, canola oil, sunflower oil | 4 | 105 | -0.68 (-0.92 to -0.44) | 21.9 | ⨁⨁◯◯  Low |
| Jolfaie 2016 | Apolipoprotein A-1 (g/L) | Rice bran oil | Peanut oil, olive oil, corn oil, canola oil, | 2 | 29 | -0.01 (-0.05 to 0.04) | 10.2 | ⨁◯◯◯  Very low |
| Jolfaie 2016 | Apolipoprotein B (g/L) | Rice bran oil | Peanut oil, olive oil, corn oil, canola oil, | 2 | 29 | -0.02 (-0.10 to 0.07) | 76.0 | ⨁◯◯◯  Very low |
| Jolfaie 2016 | Lipoprotein (mg/dL) | Rice bran oil | Peanut oil, olive oil, corn oil, canola oil, | 2 | 45 | -0.04 (-0.3 to 0.22) | 0.0 | ⨁◯◯◯  Very low |

**Supplementary Table S 7: Estimated effect sizes of coconut oil consumption on health outcomes**

| **Source** | **Outcome** | **Oil type** | **Comparator** | **No of studies** | **Sample size** | **Random effect size (95% CI)** | ***I*^2^,%** | **GRADE rating** |
| --- | --- | --- | --- | --- | --- | --- | --- | --- |
| Jayawardema 2020 | Total cholesterol  (mmol/L) | Coconut oil | Soybean oil, safflower oil, corn oil, palm oil, butter, olive oil, peanut oil, canola oil | 20 | 1,454 | 0.40 (0.23 to 0.57) | 82.5 | ⨁⨁◯◯  Low |
| Jayawardema 2020 | Triglyceride (mmol/L) | Coconut oil | Soybean oil, safflower oil, corn oil, palm oil, butter, olive oil, peanut oil, canola oil | 17 | 1,445 | 0.11 (-0.01 to 0.23) | 1.3 | ⨁⨁◯◯  Low |
| Teng 2020 | Triglycerides (mmol/L) | Coconut oil | Soybean oil, diet, safflower oil, olive oil, canola oil, palm oil, peanut oil | 12 | 532 | 0.001 (0.000 to 0.003) | 11.5 | ⨁◯◯◯  Very low |
| Duarte 2022 | Triglycerides (mmol/L) | Coconut oil | Olive oil, soybean oil, sunflower oil, chia oil, safflower oil, palm oil | 7 | 482 | -0.001 (-0.06 to 0.06) | 0 | ⨁◯◯◯  Very low |
| Jayawardema 2020 | LDL (mmol/L) | Coconut oil | Soybean oil, safflower oil, corn oil, palm oil, butter, olive oil, peanut oil, canola oil | 16 | 1,427 | 0.27 (0.12 to 0.42) | 72.8 | ⨁⨁◯◯  Low |
| Teng 2020 | LDL (mmol/L) | Coconut oil | Soybean oil, diet, safflower oil, olive oil, canola oil, palm oil, peanut oil | 12 | 532 | 0.006 (0.000 to 0.011) | 59.7 | ⨁◯◯◯  Very low |
| Duarte 2022 | LDL (mmol/L) | Coconut oil | Olive oil, soybean oil, sunflower oil, chia oil, safflower oil, palm oil | 7 | 482 | -0.05 (-0.20 to 0.10) | 79.1 | ⨁◯◯◯  Very low |
| Jayawardema 2020 | HDL (mmol/L) | Coconut oil | Soybean oil, safflower oil, corn oil, palm oil, butter, olive oil, peanut oil, canola oil | 17 | 1,445 | 0.07 (0.03 to 0.11) | 31.6 | ⨁⨁◯◯  Low |
| Teng 2020 | HDL (mmol/L) | Coconut oil | Soybean oil, diet, safflower oil, olive oil, canola oil, palm oil, peanut oil | 12 | 532 | 0.013 (0.009 to 0.017) | 6.7 | ⨁◯◯◯  Very low |
| Duarte 2022 | HDL (mmol/L) | Coconut oil | Olive oil, soybean oil, sunflower oil, chia oil, safflower oil, palm oil | 7 | 482 | 0.08 (0.01 to 0.15) | 75.4 | ⨁◯◯◯  Very low |
| Jayawardema 2020 | VLDL (mmol/L) | Coconut oil | Safflower oil, butter, corn oil, soybean oil, chia oil | 8 | 469 | 0.00 (-0.01 to 0.01) | 44.0 | ⨁◯◯◯  Very low |
| Jayawardema 2020 | LDL/HDL ratio | Coconut oil | Virgin olive oil, corn oil, soybean oil, peanut oil | 8 | 347 | 0.22 (-0.37 to 0.80) | 79 | ⨁◯◯◯  Very low |
| Jayawardema 2020 | TC/HDL ratio | Coconut oil | Soybean oil, safflower oil, virgin olive oil, butter, peanut oil, corn oil, canola oil | 5 | 363 | -0.20 (-0.34 to -0.06) | 7 | ⨁◯◯◯  Very low |
| Duarte 2022 | TC/HDL ratio | Coconut oil | Olive oil, soybean oil, chia oil, safflower oil | 3 | 160 | -0.06 (-0.44 to 0.32) | 81.7 | ⨁◯◯◯  Very low |
| Jayawardema 2020 | TG/HDL ratio | Coconut oil | Safflower oil, chia oil, soybean oil | 1 | 135 | 0.11 (-0.34 to 0.57) | 62 | ⨁◯◯◯  Very low |
| Jayawardema 2020 | Apolipoprotein A-1 (g/L) | Coconut oil | Safflower oil, butter, soybean oil, peanut oil | 3 | 202 | 0.001 (0.006 to 0.002) | 0 | ⨁◯◯◯  Very low |
| Jayawardema 2020 | Apolipoprotein B (g/L) | Coconut oil | Safflower oil, butter, soybean oil, peanut oil | 3 | 202 | -0.0001 (-0.002 to 0.002) | 44 | ⨁◯◯◯  Very low |
| Jayawardema 2020 | HbA1c (%) | Coconut oil | Soybean oil, safflower oil, sunflower oil, chia oil | 2 | 301 | -0.39 (-0.50 to -0.27) | 31.7 | ⨁◯◯◯  Very low |
| Jayawardema 2020 | Fasting blood sugar (mmol/L) | Coconut oil | Soybean oil, palm oil, butter, olive oil, peanut oil | 5 | 235 | 0.04 (-0.15 to 0.06) | 0 | ⨁◯◯◯  Very low |
| Duarte 2022 | Fasting blood sugar (mmol/L) | Coconut oil | Olive oil, soybean oil | 4 | 212 | 0.06 (-0.05 to 0.18) | 0 | ⨁◯◯◯  Very low |
| Dhanasekara 2022 | Fasting blood sugar (mmol/L) | Coconut oil | Soybean oil, olive oil, palm oil, sea buckthorn berry oil, butter, peanut oil, sesame oil | 10 | 444 | 2.05 (-0.14 to 4.25) | 6.6 | ⨁⨁◯◯  Low |
| Dhanasekara 2022 | Fasting insulin | Coconut oil | Soybean oil, palm oil, peanut oil | 5 | 163 | 0.31 (-2.59 to 3.20) | 28.0 | ⨁◯◯◯  Very low |
| Dhanasekara 2022 | HOMA-IR | Coconut oil | Soybean oil, palm oil, peanut oil | 5 | 163 | 0.55 (0.00 to 1.10) | 63.8 | ⨁◯◯◯  Very low |
| Dhanasekara 2022 | HOMA-β | Coconut oil | Soybean oil, palm oil, peanut oil, corn oil | 6 | 209 | -17.15 (-44.36 to 10.09) | 82.2 | ⨁◯◯◯  Very low |
| Jayawardema 2020 | c-reactive protein (mg/L) | Coconut oil | Sunflower oil, palm oil, olive oil | 3 | 155 | -0.15 (-0.72 to 0.43) | 3 | ⨁◯◯◯  Very low |
| Neelathakan 2020 | c-reactive protein (mg/L) | Coconut oil | Soybean oil, peanut oil, olive oil, corn oil | 5 | 188 | -0.003 (-0.85 to 0.85) | 53.7 | ⨁◯◯◯  Very low |
| Duarte 2022 | c-reactive protein (mg/L) | Coconut oil | Olive oil, soybean oil | 2 | 131 | -0.36 (-1.15 to 1.87) | 74.6 | ⨁◯◯◯  Very low |
| Neelathakan 2020 | Body weight (kg) | Coconut oil | Soybean oil, safflower oil, peanut oil, olive oil, corn oil | 8 | 456 | -0.23 (-0.81 to 0.35) | 62.7 | ⨁◯◯◯  Very low |
| Duarte 2022 | Body weight (kg) | Coconut oil | Olive oil, soybean oil, sunflower oil, chia oil | 6 | 409 | -0.39 (-1.19 to 0.41) | 81.0 | ⨁◯◯◯  Very low |
| Swarnamali  2023 | Body weight (kg) | Coconut oil | Safflower oil, olive oil, peanut oil, chia oil, soybean oil, sunflower oil, mixed oil | 7 | 449 | -0.51 (-1.22 to 0.20) | 69.9 | ⨁◯◯◯  Very low |
| Jayawardema 2020 | Body mass index (kg/m^2^) | Coconut oil | Soybean oil, peanut oil | 3 | 88 | -0.04 (-1.38 to 1.29) | 0 | ⨁◯◯◯  Very low |
| Swarnamali  2023 | Body mass index (kg/m^2^) | Coconut oil | Safflower oil, olive oil, peanut oil, chia oil, soybean oil, sunflower oil, mixed oil | 7 | 534 | 0.37 (-0.10 to 0.84) | 93.7 | ⨁◯◯◯  Very low |
| Neelathakan 2020 | Waist circumference (cm) | Coconut oil | Soybean oil, safflower oil, olive oil, peanut oil | 4 | 131 | -0.63 (-2.44 to 1.19) | 42.7 | ⨁◯◯◯  Very low |
| Duarte 2022 | Waist circumference (cm) | Coconut oil | Olive oil, soybean oil, chia oil, safflower oil | 5 | 240 | -0.83 (-1.89 to 0.24) | 80.0 | ⨁◯◯◯  Very low |
| Swarnamali  2023 | Waist circumference (cm) | Coconut oil | Safflower oil, olive oil, peanut oil, chia oil, soybean oil, sunflower oil, mixed oil | 7 | 289 | -0.83 (-1.98 to 0.32) | 85.1 | ⨁◯◯◯  Very low |
| Swarnamali  2023 | Waist hip ratio | Coconut oil | Safflower oil, chia oil, soybean oil, sunflower oil | 3 | 294 | -0.01 (-0.02 to 0.01) | 96.7 | ⨁◯◯◯  Very low |
| Neelathakan 2020 | Body fat (%) | Coconut oil | Sunflower oil, safflower oil, olive oil, peanut oil | 5 | 206 | 0.03 (-0.33 to 0.38) | 0 | ⨁◯◯◯  Very low |
| Duarte 2022 | Body fat (%) | Coconut oil | Olive oil, sunflower oil, soybean oil, chia oil, safflower oil | 5 | 445 | -0.10 (-0.61 to 0.42) | 78.2 | ⨁◯◯◯  Very low |
| Swarnamali  2023 | Body fat (%) | Coconut oil | Safflower oil, olive oil, peanut oil, chia oil, soybean oil, sunflower oil | 6 | 394 | -0.38 (-0.67 to -0.09) | 48.1 | ⨁◯◯◯  Very low |
| Swarnamali  2023 | Body fat (kg) | Coconut oil | Safflower oil, peanut oil, mixed oil | 3 | 86 | -0.25 (-0.73 to 0.22) | 0 | ⨁⨁◯◯  Low |
| Dhanasekara 2022 | Area under curve of glucose | Coconut oil | Yoghurt, olive oil | 6 | 253 | 182.48 (2.73 to 322.22) | 82.2 | ⨁◯◯◯  Very low |
| Dhanasekara 2022 | Area under curve of insulin | Coconut oil | Yoghurt, olive oil | 5 | 201 | -459.13 (-899.99 to -28.27) | 89.9 | ⨁◯◯◯  Very low |

**Supplementary Table S 8: Estimated effect sizes of palm oil consumption on health outcomes**

| Source | Outcome | Oil type | Comparator | No of studies | Sample size | Random effect size (95% CI) | *I*^2^,% | GRADE rating |
| --- | --- | --- | --- | --- | --- | --- | --- | --- |
| Fattore 2014 | Total cholesterol (mmol/L) | Palm oil | Peanut oil, olive oil, canola oil, soybean oil, sunflower oil, corn oil | 16 | 1019 | 0.24 (0.06 to 0.42) | 75.5 | ⨁⨁◯◯  Low |
| Sun 2015 | Total cholesterol (mmol/L) | Palm oil | Peanut oil, olive oil, canola oil, soybean oil, sunflower oil, corn oil | 27 | 730 | 0.35 (0.23 to 0.47) | 86.0 | ⨁⨁◯◯  Low |
| Wang 2019 | Total cholesterol (mmol/L) | Palm oil | Olive oil, canola oil, sunflower oil, peanut oil, soybean oil, soyabean oil, corn oil | 11 | 627 | -0.01 (-0.08 to 0.07) | 26.0 | ⨁⨁◯◯  Low |
| Hisham 2020 | Total cholesterol (mmol/L) | Palm oil | Soybean oil, peanut oil, safflower oil, sunflower oil, olive oil, canola oil, | 19 | 580 | 0.30 (0.13 to 0.47) | 65.8 | ⨁◯◯◯  Very low |
| Fattore 2014 | Triglyceride (mmol/L) | Palm oil | NR | 15 | 929 | 0.01 (-0.08 to 0.12) | 60.0 | ⨁◯◯◯  Very low |
| Sun 2015 | Triglyceride  (mmol/L) | Palm oil | Peanut oil, olive oil, canola oil, soybean oil, sunflower oil, corn oil | 25 | 657 | -0.02 (-0.08 to 0.05) | 63.4 | ⨁⨁◯◯  Low |
| Wang 2019 | Triglycerides (mmol/L) | Palm oil | Olive oil, canola oil, sunflower oil, peanut oil, soybean oil, soyabean oil, corn oil | 11 | 767 | 0.00 (-0.07 to 0.08) | 77.0 | ⨁◯◯◯  Very low |
| Hisham 2020 | Triglyceride  (mmol/L) | Palm oil | Soybean oil, peanut oil, safflower oil, sunflower oil, olive oil, canola oil, | 19 | 580 | 0.02 (-0.05 to 0.09) | 0 | ⨁◯◯◯  Very low |
| Fattore 2014 | LDL (mmol/L) | Palm oil | Peanut oil, olive oil, canola oil, soybean oil, sunflower oil, corn oil | 19 | 1493 | 0.26 (0.08 to 0.44) | 62.9 | ⨁⨁◯◯  Low |
| Sun 2015 | LDL (mmol/L) | Palm oil | Peanut oil, olive oil, canola oil, soybean oil, sunflower oil, corn oil | 26 | 716 | 0.24 (0.13 to 0.35) | 83.1 | ⨁⨁◯◯  Low |
| Wang 2019 | LDL (mmol/L) | Palm oil | Olive oil, canola oil, sunflower oil, peanut oil, soybean oil, soyabean oil, corn oil | 11 | 961 | -0.05 (-0.11 to 0.01) | 21.1 | ⨁⨁◯◯  Low |
| Hisham 2020 | LDL (mmol/L) | Palm oil | Soybean oil, peanut oil, safflower oil, sunflower oil, olive oil, canola oil, | 19 | 580 | 0.30 (0.08 to 0.51) | 83.0 | ⨁◯◯◯  Very low |
| Fattore 2014 | HDL (mmol/L) | Palm oil | Peanut oil, olive oil, canola oil, soybean oil, sunflower oil, corn oil | 19 | 1469 | 0.28 (0.05 to 0.50) | 75.2 | ⨁⨁◯◯  Low |
| Sun 2015 | HDL (mmol/L) | Palm oil | Peanut oil, olive oil, canola oil, soybean oil, sunflower oil, corn oil | 26 | 716 | 0.03 (0.01 to 0.04) | 48.8 | ⨁⨁◯◯  Low |
| Wang 2019 | HDL (mmol/L) | Palm oil | Olive oil, canola oil, sunflower oil, peanut oil, soybean oil, soyabean oil, corn oil | 11 | 767 | 0.04 (-0.03 to 0.11) | 92.7 | ⨁◯◯◯  Very low |
| Hisham 2020 | HDL (mmol/L) | Palm oil | Soybean oil, peanut oil, safflower oil, sunflower oil, olive oil, canola oil, | 19 | 580 | 0.06 (0.03 to 0.10) | 0 | ⨁◯◯◯  Very low |
| Fattore 2014 | vLDL (mg/dL) | Palm oil | NR | 4 | 193 | -0.03 (-0.03 to 0.08) | 0 | ⨁◯◯◯  Very low |
| Fattore 2014 | LDL/HDL ratio | Palm oil | NR | 2 | 108 | 0.21 (-0.05 to 0.47) | 0 | ⨁◯◯◯  Very low |
| Fattore 2014 | Apolipoprotein A-1 (g/L) | Palm oil | NR | 7 | 523 | 0.07 (0.04 to 0.11) | 0 | ⨁◯◯◯  Very low |
| Hisham 2020 | Apolipoprotein A-1 (g/L) | Palm oil | Canola oil, soybean oil, sunflower oil, olive oil | 5 | 155 | 0.33 (-1.38 to 2.04) | 5.2 | ⨁◯◯◯  Very low |
| Fattore 2014 | Apolipoprotein B (g/L) | Palm oil | NR | 7 | 523 | 0.05 (0.02 to 0.08) | 0 | ⨁◯◯◯  Very low |
| Hisham 2020 | Apolipoprotein B (g/L) | Palm oil | Canola oil, soybean oil, sunflower oil, olive oil | 5 | 155 | 0.09 (-0.01 to 0.19) | 0 | ⨁◯◯◯  Very low |
| Fattore 2014 | TC/HDL | Palm oil | NR | 5 | 498 | -0.19 (-0.43 to 0.06) | 22.7 | ⨁◯◯◯  Very low |
| Fattore 2014 | Lipoprotein A (g/L) | Palm oil | NR | 2 | 108 | -0.01 (-0.14 to 0.10) | 0 | ⨁◯◯◯  Very low |
| Dong 2017 | Body weight (kg) | Red palm oil | Peanut oil, sunflower oil | 4 | 988 | 0.05 (-0.08 to 0.17) | 0 | ⨁◯◯◯  Very low |
| Zulkiply 2019 | Fasting plasma glucose (mmol/L) | Palm oil | Soybean oil, olive oil | 5 | 211 | 0.01 (-0.09 to 0.10) | 14.1 | ⨁◯◯◯  Very low |
| Zulkiply 2019 | Fasting insulin (pmol/L) | Palm oil | Soybean oil, olive oil | 4 | 177 | 0.13 (-4.27 to 4.54) | 0 | ⨁◯◯◯  Very low |

**Supplementary Table S 9: Estimated effect sizes of palm olein consumption on health outcomes**

| Source | Outcome | Oil type | Comparator | No of studies | Sample size | Random effect size (95% CI) | *I*^2^,% | GRADE rating |
| --- | --- | --- | --- | --- | --- | --- | --- | --- |
| Voon 2019 | Total cholesterol (mmol/L) | Palm olein | Canola oil, coconut oil, soybean oil, sunflower oil, peanut oil, olive oil | 9 | 1,075 | -0.10 (-0.30 to 0.10) | 78.7 | ⨁◯◯◯  Very low |
| Voon 2019 | LDL (mmol/L) | Palm olein | Canola oil, coconut oil, soybean oil, sunflower oil, peanut oil, olive oil | 9 | 1,075 | -0.06 (-0.29 to 0.17) | 84.7 | ⨁◯◯◯  Very low |
| Voon 2019 | HDL (mmol/L) | Palm olein | Canola oil, coconut oil, soybean oil, sunflower oil, peanut oil, olive oil | 9 | 1,075 | 0.01 (-0.03 to 0.06) | 59.2 | ⨁◯◯◯  Very low |
| Voon 2019 | Triglyceride (mmol/L) | Palm olein | Canola oil, coconut oil, soybean oil, sunflower oil, peanut oil, olive oil | 9 | 1,075 | 0.02 (-0.10 to 0.14) | 0 | ⨁◯◯◯  Very low |
| Voon 2019 | TC/HDL ratio | Palm olein | Canola oil, coconut oil, soybean oil, sunflower oil, peanut oil, olive oil | 5 | 647 | -0.15 (-0.43 to 0.14) | 64.1 | ⨁◯◯◯  Very low |

**Supplementary Table S 10: Estimated effect sizes of sesame oil consumption on health outcomes**

| Source | Outcome | Oil type | Comparator | No of studies | Sample size | Random effect size (95% CI) | *I*^2^,% | GRADE rating |
| --- | --- | --- | --- | --- | --- | --- | --- | --- |
| Atefi 2022 | HbA1c (%) | Sesame oil | Soybean oil, mixed oil | 4 | 106 | -2.06 (-3.47 to -0.65) | 95.0 | ⨁◯◯◯  Very low |
| Atefi 2022 | Fasting blood sugar (mmol/L) | Sesame oil | Soybean oil, coconut oil, mixed oil | 5 | 643 | -0.18 (-0.26 to -0.10) | 97.9 | ⨁◯◯◯  Very low |
| Atefi 2022 | Serum insulin (μU/L) | Sesame oi | Soybean oil, sunflower oil, | 3 | 562 | 3.18 (-1.28 to 7.63) | 99.3 | ⨁◯◯◯  Very low |
| Atefi 2022 | Systolic blood pressure (mmHg) | Sesame oil | Soybean oil, mixed oil | 5 | 485 | -2.68 (-5.26 to -0.10) | 99.2 | ⨁◯◯◯  Very low |
| Atefi 2022 | Diastolic blood pressure (mmHg) | Sesame oil | Soybean oil, mixed oil | 5 | 485 | -1.98 (-3.92 to -0.05) | 98.9 | ⨁◯◯◯  Very low |
| Atefi 2022 | Malondialdehyde (nmol/dL) | Sesame oil | Soybean oil, sunflower oil, mixed oil | 5 | 539 | -4.86 (-6.75 to -2.98) | 98.0 | ⨁◯◯◯  Very low |
| Atefi 2022 | Body weight (kg) | Sesame oil | Sunflower oil, olive oil, mixed oil | 5 | 613 | -0.35 (-0.61 to -0.05) | 86.2 | ⨁◯◯◯  Very low |
| Atefi 2022 | Body mass index (kg/m^2^) | Sesame oil | Sunflower oil, mixed oil | 5 | 613 | -0.38 (-0.72 to -0.05) | 88.4 | ⨁◯◯◯  Very low |
